# Predicting progression events in multiple myeloma from routine blood work

**DOI:** 10.1038/s41746-025-01636-9

**Published:** 2025-04-30

**Authors:** Maximilian Ferle, Nora Grieb, Markus Kreuz, Jonas Ader, Hartmut Goldschmidt, Elias K. Mai, Uta Bertsch, Uwe Platzbecker, Thomas Neumuth, Kristin Reiche, Alexander Oeser, Maximilian Merz

**Affiliations:** 1 Center for Scalable Data Analytics and Artificial Intelligence (ScaDS.AI) Dresden/Leipzig, Universität Leipzig, Leipzig, Germany; 2 Innovation Center Computer Assisted Surgery (ICCAS), University of Leipzig, Leipzig, Germany; 3 Department of Medical Bioinformatics, Fraunhofer Institute for Cell Therapy and Immunology, Leipzig, Germany; 4 Department of Hematology, Hemostaseology, Cellular Therapy and Infectiology, University Hospital of Leipzig, Leipzig, Germany; 5 Department of Internal Medicine V, University Hospital Heidelberg, Heidelberg, Germany; 6 National Center for Tumor Diseases (NCT), Heidelberg, Germany; 7 Institute for Clinical Immunology, University Hospital of Leipzig, Leipzig, Germany; 8 Synagen GmbH, Dresden, Germany; 9 Multiple Myeloma and Transplant and Cellular Therapy Services, Department of Medicine, Memorial Sloan Kettering Cancer Center, New York, NY USA

**Keywords:** Myeloma, Experimental models of disease, Cancer models

## Abstract

This study introduces a system for predicting disease progression events in multiple myeloma patients from the CoMMpass study (*N* = 1186). Utilizing a hybrid neural network architecture, our model predicts future blood work from historical lab results with high accuracy, significantly outperforming baseline estimators for key disease parameters. Disease progression events are annotated in the forecasted data, predicting these events with significant reliability. We externally validated our model using the GMMG-MM5 study dataset (*N* = 504), and could reproduce the main results of our study. Our approach enables early detection and personalized monitoring of patients at risk of impeding progression. Designed modularly, our system enhances interpretability, facilitates integration of additional modules, and uses routine blood work measurements to ensure accessibility in clinical settings. With this, we contribute to the development of a scalable, cost-effective virtual human twin system for optimized healthcare resource utilization and improved outcomes in multiple myeloma patient care.

## Introduction

Multiple Myeloma (MM) is a heterogeneous disease with survival ranging from months to decades based on a patient’s individual risk profile^1^. Characteristics associated with outcomes include disease-specific and patient-derived factors, as well as treatment-associated features, like the emergence of side effects and access to latest treatment innovations. In the past, several scoring systems have been implemented to estimate the prognosis of MM patients with newly diagnosed multiple myeloma (NDMM). While the initial version of the International Staging System (ISS)^2^ accounted for factors associated with disease burden, the first (R-ISS)^3^ and second revisions (R2-ISS)^4^ of the ISS integrated cytogenetic data and lactate dehydrogenase to appreciate disease biology and aggressiveness. More lately, clinical, demographic, genomic, and therapeutic data^5^ as well as immune signatures have been used to assign patients into different prognostic risk categories^6^. However, the majority of those established scoring systems solely rely on the initial assessment of the patient. Longitudinal changes upon treatment initiation, like deterioration of laboratory values or disease kinetics are usually not accounted for. According to current guidelines, patients with MM undergoing treatment for newly diagnosed or relapsed disease should be followed-up at least every 12 weeks^7^. These routine visits should include a clinical assessment and laboratory testing with a complete blood count, renal/liver function testing as well as myeloma-specific markers of disease activity. The regular monitoring and quarterly lab testing for MM patients produces an extensive collection of longitudinal data, presenting a significant challenge for analysis with traditional methods for survival analysis. This wealth of data creates a growing need for more advanced processing techniques that can streamline the integration of this data, facilitate the identification of trends, predict patient outcomes, and optimize individualized care plans. In recent years, neural network (NN) based models, specifically Long Short-Term Memory (LSTM) networks^8^, were shown to be effective in analyzing and modeling disease trajectories in a variety of cancer types, outperforming various other methods for time-series analysis. Here, applications ranged from the detection of brain tumors based on sequential magnetic resonance images^9^, predicting survival outcomes in prostate cancer from longitudinal clinicopathological data^10^, using time-series tumor marker data for early detection of several types of cancer^11^ to predicting cancer symptom evolution on the basis of past routinely collected nursing documentation^12^. However, the use of sophisticated machine learning (ML) techniques to predict patient outcomes in MM is still in its infancy. A very recent review by Allegra et al. ^13^ summarized the latest advances in utilizing ML techniques for diagnosis, prognosis, and treatment selection based on clinical and gene expression data. Therein, it is described how random forests^14–17^, clustering and graph-based approaches^15–18^ as well as NNs^15,16^ can predict survival outcomes based on treatment, clinical and expression data. However, these applications rely on stationary (mostly initial) assessments and do not account for longitudinal kinetics. To the best of our knowledge, no prior study investigated the potential of leveraging the wealth of longitudinal data that accumulate in routine MM care. In this study, we introduce a predictive framework for analyzing individual disease trajectories of MM patients and, subsequently, the prediction of individual progression events. Our study demonstrates how ML can be used to individualize and periodically refine risk assessment based on existing routine laboratory values. These findings could play a crucial role in dynamically refining follow-up schedules or introducing preventative treatments for patients who are at an impending risk of future disease progression. This approach aligns with the vision of the vitural human twin (VHT) that facilitates the development and validation of patient-specific predictive models, thereby enhancing clinical decision support and personalized health forecasting^19^^,^^20^.

## Results

Our study aimed to develop and validate a model capable of generating likely future blood work for MM patients based on the patients unique previous trajectories (Forecasting Model; see methods sections Long Short-Term Memory Conditional Restricted Boltzmann Machine & Forecasting Model). Following this, we aimed to evaluate the predictive utility of these forecasts against a critical clinical endpoint to determine their effectiveness in anticipating such outcomes. Given its significant clinical implications, progressive disease (PD) as defined by the International Myeloma Working Group (IMWG) consensus criteria^21^ was selected as the primary endpoint of interest. To this end, we have implemented a progression prediction metamodel, integrating our forecasting model with a progression annotation model adept at identifying and annotating progression events within MM disease trajectories (Annotation Model; see methods section Annotation Model). This enabled the anticipation of progression events well before they would otherwise become apparent. The efficacy of the interplay of the two models was assessed through a series of analyses, the results of which are detailed below.

### Forecasting model performance

**Fig. 1:**
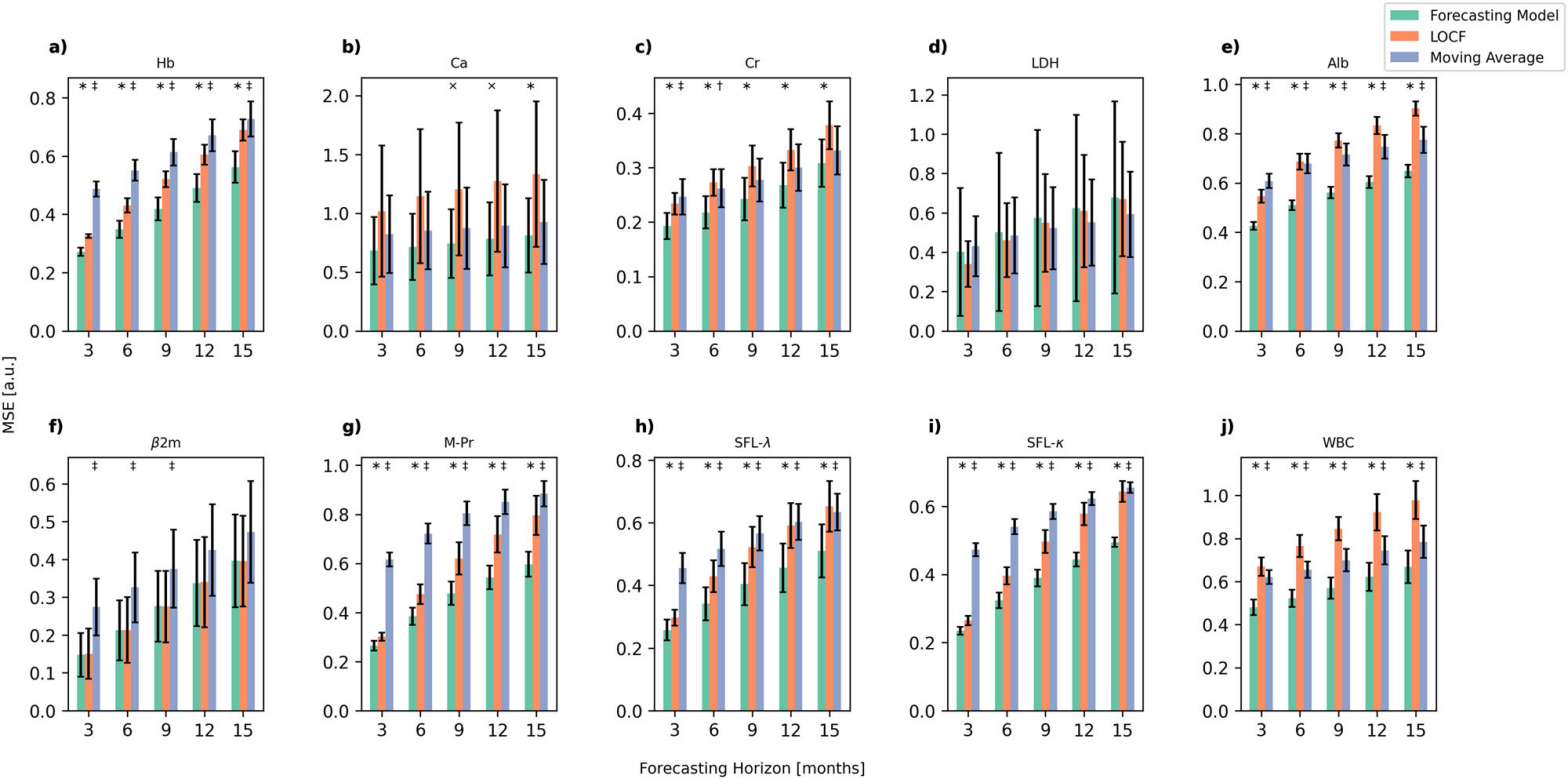
Comparison of forecasting model accuracy across different horizons. Mean squared error (MSE) of three different forecasting approaches across various forecasting horizons. Error bars represent the standard deviation of the MSE obtained from cross-validation folds. The forecasting model (shown in green), last observation carried forward (LOCF, shown in orange), and moving average (MA, shown in blue) are compared. Each panel represents a different blood work parameter: (**a**) Hemoglobin (Hb), (**b**) Calcium (Ca), (**c**) Creatinine (Cr), (**d**) Lactate Dehydrogenase (LDH), (**e**) Albumin, (**f**) Beta-2-Microglobulin (β2m), (**g**) M-Protein (M-Pr), (**h**) Serum free light-chain lambda (SFL-λ), (**i**) Serum free light-chain kappa (SFL-κ), (**j**) White blood cells (WBC). The forecasting model consistently shows lower MSE compared to LOCF and MA for all parameters except LDH. Statistical significance is indicated by a cross and asterisk for comparisons between the forecasting model and LOCF, and a dagger and double dagger for comparisons between the forecasting model and MA, corresponding to *p*-values less than *p* < 0.1 and *p* < 0.05 respectively, based on a one-sided Mann-Whitney *U* test.

**Fig. 2:**
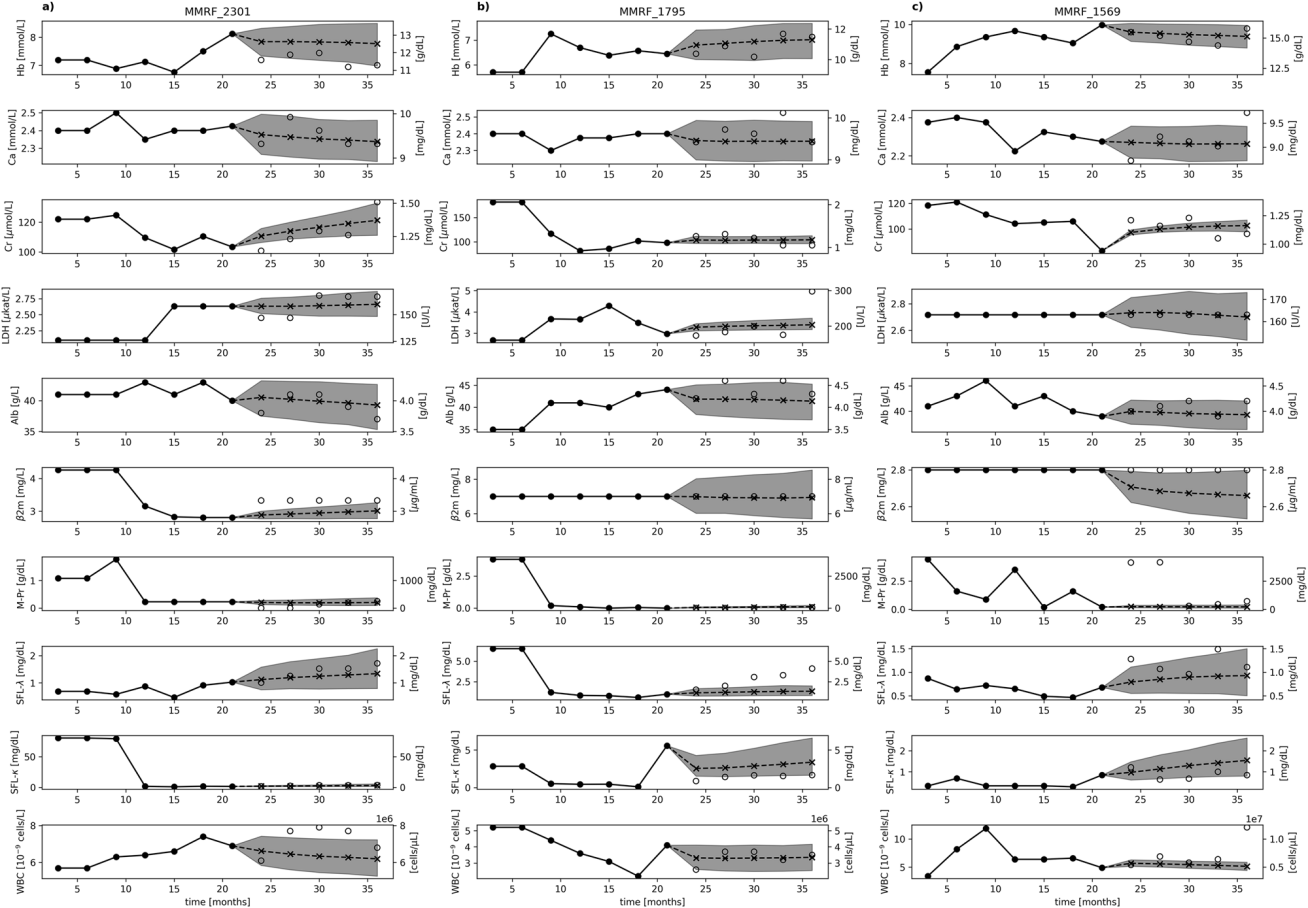
Forecasted patient trajectories under different myeloma treatments. Forecasted patient trajectories for three individual patients undergoing different treatments. The patients were treated with (**a**) Bortezomib-based, (**b**) Carfilzomib-based, and (**c**) IMIDs-based. The forecasting model utilized the initial seven follow-ups, equivalent to 21 months of clinical data, to predict the subsequent five follow-ups, which span an additional 15 months. Dashed lines and crosses represent the forecasts, while circles denote actual observations. Gray sleeves illustrate the 95% confidence interval of the distribution of forecasted trajectories.

To evaluate the model’s accuracy in forecasting blood work, we calculated its mean squared error (MSE) for individual parameters and compared it to naive baseline estimators using Last Observation Carried Forward (LOCF) and moving average (MA) across different forecasting horizons (Fig. 1). The forecasting model consistently outperformed the baseline estimators for all parameters except Lactate dehydrogenase (LDH) (Fig. 1d) across all forecasting horizons. Notably, this superior performance was statistically significant for the key disease parameters M-Protein (M-Pr), serum free light-chain λ (SFL-λ), and serum free light-chain κ (SFL-κ) (p(LOCF) < 0.05; p(MA) < 0.05; Figs. 1g–i). Similar significance was observed for other parameters such as Hemoglobin (Hb) (Fig. 1a), Albumin (Alb) (Fig. 1e), and White blood bells (WBC) (Fig. 1j), as well as Creatinine (Cr) in the short term, with both LOCF and MA comparisons yielding p < 0.05. To assess the model’s performance in predicting sudden changes in disease parameters, we conducted a focused analysis on prediction errors under conditions of extreme variability. Specifically, we isolated instances where changes in parameters exceeded one standard deviation of all changes, representing the most extreme 31.8% of changes observed in our dataset. This subset was analyzed to evaluate the model’s ability to handle abrupt shifts in clinical markers, which are critical in the management of multiple myeloma. Our findings, illustrated in Supplementary Fig. 1, demonstrate that even in these challenging scenarios, the model significantly outperforms the baseline estimators for the disease parameters M-Pr, SFL-λ and SFL-κ (p < 0.05). These findings lend credibility to the model’s potential utility in clinical settings, where rapid changes in patient condition must be accurately addressed. The individual features in the model’s prediction exhibited strong correlations with the actual patient data (Supplementary Table 1). We thus extended our analysis to examine the correlations of the blood work for predictions across multiple future follow-ups and compare those produced by our model to those produced by the baseline estimators. Here, we found that all models produced comparably high correlations, with coefficients ranging from *r* = 0.34 ± 0.08 (Calcium (Ca) at the 15 months forecast interval) to *r* = 0.92 ± 0.02 (β-2-microglobulin (β2m) at the 3 months forecast interval). We attribute this to the fact that the variability of blood work parameters was found to be greater between patients than wihtin a single patient, resulting to an artificially high correlation when the forecasted value was of similar magnitude to the last observed value. However, the correlations produced by our model were always equal or greater than those produced by the baseline estimators at all forecasting horizons for all parameters except LDH after the 3 months interval (outperformed by LOCF), β2m at any timepoint (outperformed by LOCF) and Cr at the 15 months interval (outperformed by LOCF) (Supplementary Table 2). Based on this, we realized that most features display strong inertia. We hence assessed the ability of our model to capture data trends by comparing the differences between the last known values and our model’s forecasts (∆_model_) with the differences between the last known values and the actual subsequent observations (∆_true_). As this trivially results in a correlation of *r* = 0 for LOCF and the generally poor performance of MA, we excluded the baseline predictors from this analysis. Here, moderate correlations were found (0.62 ± 0.04 ≥ r ≥ 0.25 ± 0.03) throughout all features in the blood work, indicating that the model was correctly forecasting the momentum of the data. The smallest r-value recorded was *r* = 0.25 ± 0.03 for β2m at 6 and 9 months forecast interval respectively, while the largest r-value was *r* = 0.62 ± 0.04 for Ca at 15 months forecast. All reported correlation values were significant. Initial evaluations of the forecasting model’s outputs revealed a high degree of accuracy (R^2^ = 0.81 ± 0.03) in reflecting the cross-correlations among the ten key features of the blood work derived from the ground truth patient data (see methods section Training and Internal Validation Patient Characteristics), as shown in the correlation matrix of Supplementary Fig. 2a. Supplementary Fig. 2b specifically focuses on comparing correlation coefficients. It presents a scatterplot that juxtaposes the coefficients from the actual data with those obtained from the forecasted data across varying forecasting horizons. Here, horizons ranged from 3–15 months, increasing in 3 month increments. It is important to note that the model’s ability to predict correlations accurately was strongest at the shortest horizon of three months and diminished with longer horizons. For conciseness, this trend was aggregated in Supplementary Fig. 2b, to reflect the model’s overall performance across all studied forecasting horizons. Importantly, clinically relevant correlations like the positive correlation between serum Cr and β2m levels or the inverse relationship between serum free light-chain (SFL) types and Alb were reproduced. In contrast, Supplementary Fig. 2c explores the temporal dynamics of the time series data by examining its lagged autocorrelation, a key statistical property. This Figure compares the lag-correlation coefficients for each pair of features in the observed data versus the forecasted data at five distinct lag times: 3, 6, 9, 12, and 15 months. Each panel within Supplementary Fig. 2c provides a detailed view of the lag-correlations for each of the considered lag times. The results demonstrate high accuracy in reproducing the autocorrelation of the time series, with R^2^ values decreasing with increasing lag times: R^2^ = 0.95 ± 0.01 for a 3 months lag, R^2^ = 0.92 ± 0.00 for a 6 months lag, R^2^ = 0.89 ± 0.01 for a 9 months lag, R^2^ = 0.86 ± 0.02 for a 12 months lag, and R^2^ = 0.83 ± 0.03 for a 15 months lag. These findings underscore the model’s robustness in capturing the temporal dependencies between features, with a decrease in accuracy as the lag time increases. In Fig. 2, we present the predicted trajectories for three randomly selected patients undergoing Bortezomib- (Fig. 2a) Carfilzomib- (Fig. 2b) and IMIDs-based (Fig. 2c) first-line treatments. The forecasting model was provided with the initial seven follow-ups, corresponding to 21 months of clinical data, to forecast the subsequent five follow-ups, covering an additional 15 months. Note that the forecasting model was not provided with information about the ongoing treatment and thus forecasted future blood work data merely based on the kinetics of the patients’ unique trajectories. To gain a deeper understanding of the forecasting model’s internal mechanisms, we additionally conducted a SHapley Additive exPlanation (SHAP) value analysis (see methods section SHAP Value Computation). This approach enabled us to approximately quantify each feature’s contribution to the model’s prediction for another feature and thus explore interactions between features. Each scatter plot in Supplementary Fig. 4 corresponds to a pairwise interaction between two features, with one feature’s input values on the x-axis and the SHAP values for the corresponding feature (impact on the model’s output) on the y-axis. Supplementary Fig. 4a displays the impact an input feature value has on the prediction of itself, while Supplementary Fig. 4b illustrates the pairwise interactions for the blood work features included in the model. Here, we found that the impact an input feature had on the prediction of itself was always one order of magnitude greater than the interaction between features (Supplementary Fig. 4a, b). From these findings, we inferred that the model primarily bases its predictions for specific features on their historical levels, and further refines these predictions by integrating the interactions with other features. Notably, a strong negative interaction is observed between SFL-κ and SFL-λ, where high values of one are associated with low values of the other, reflecting their well-known clinical relationship. Positive interactions are evident between SFL and Cr, where higher levels of SFL drive an increase in Cr, and between β2m and Cr, where elevations in either biomarker are mutually associated with increases in the other. In contrast, a strong negative interaction is observed between SFL and Alb, with higher SFL values associated with lower Alb levels. These patterns align with clinical expectations, as the positive relationships between SFL and Cr, as well as β2m and Cr, are reflective of their respective roles in renal dysfunction and disease progression. Similarly, elevated SFL levels are often associated with declining albumin levels due to systemic inflammation or impaired protein synthesis. These findings not only enhance the interpretability of our model but also demonstrate its ability to capture clinically relevant relationships between biomarkers, reinforcing confidence that the predictions are both biologically meaningful and aligned with established medical knowledge.

### Progression annotation performance

**Fig. 3:**
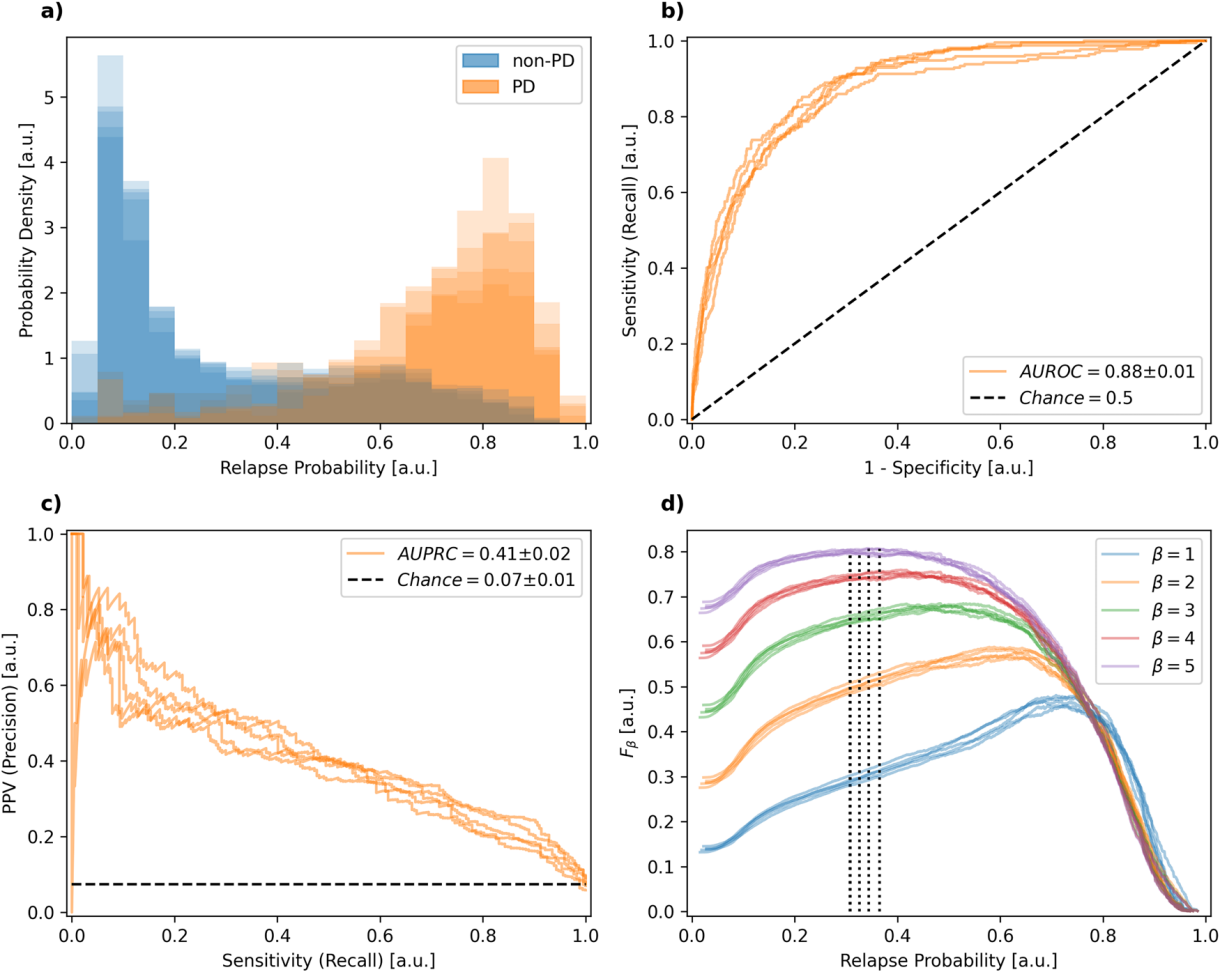
Annotation model performance across different metrics. Annotation model performance data. The data includes (**a**) probability density functions (PDF) for the output probabilities of the annotation model, color-coded for PD (progressive disease, orange) and non-PD instances (blue). PDFs from the five cross-validation folds are overlaid with 20% opacity to visually represent the consistency across models. **b** Receiver Operating Characteristic (ROC) curves of the annotation model across cross-validation folds. The area under the ROC curve (AUROC) equals 0.88 ± 0.01. **c** Precision-Recall Curve (PRC) of the annotation model across cross-validation folds. The area under the PRC (AUPRC) equals 0.41 ± 0.02. **d** F_β_ curves for *β* values of 1 through 5. Dotted lines indicate the relapse probability cut-off value of 0.33 ± 0.02, yielding an optimal F_5_ value of 0.80 ± 0.00. All values are reported as mean ± standard deviation across cross-validation folds.

Motivated by the need to accurately flag progression events in forecasted blood work, we enhanced our prediction model with a module specifically trained to output probabilities of progression based on a time series of blood work features. We coined this module Annotation Model (see methods section Annotation Model). In Fig. 3a, the probability density function (PDF) for the output probabilities of this annotation model is displayed, color-coded for PD and non-PD instances. The PDFs from the five cross-validation folds are overlaid with 20% opacity, allowing the combined opacity to visually represent the consistency across models. The annotation model’s initial performance was quantified using the area under the receiver operating characteristic curve (AUROC), yielding a robust score of 0.88 ± 0.01 (Fig. 3b), indicating effective discrimination between PD and non-PD instances in the blood work time series data. Given the low prevalence of progression events of 7% ± 1% (see Table 2 and methods Annotation Model), we also considered the precision-recall curve (PRC), depicted in Fig. 3c. The area under the PRC (AUPRC) was found to be 0.41 ± 0.02, suggesting an average positive predictive value (PPV) of 41% across all potential decision thresholds, significantly exceeding chance level of 7%. To determine an optimal decision threshold while addressing the low prevalence issue, we optimized the F_β_ score, which is defined as follows^22^:

1 $${F}_{\beta }=\left(1+{\beta }^{2}\right)\cdot \frac{{precision}\cdot {recall}}{\left({\beta }^{2}\cdot {precision}\right)+{recall}}$$

Figure 3d presents the F_β_ curves for β values of 1 through 5. We chose a β of 5, thereby emphasizing recall over precision by a factor of five, leading to a decision threshold of 0.33 ± 0.02 and an F_5_ of 0.80 ± 0.00 according to Eq. (1). The application of this optimized decision threshold to the testing data yielded a sensitivity of 0.92 ± 0.02 and a specificity of 0.65 ± 0.03 (Table 1). Our choice for β = 5 was motivated by prioritizing higher sensitivity while accepting a higher false positive rate and lower specificity as a trade-off since missing a progression event would have more negative clinical relevance than indicating a progression event where there is none. It is crucial to note that the F_β_ optimization was conducted on the training data to avoid an overly optimistic bias in the model’s performance when applied to the testing data.

**Table 1:** Annotation model baseline performance

AUROC	AUPRC	Sensitivity	Specificity
0.88 ± 0.01	0.41 ± 0.02	0.92 ± 0.02	0.65 ± 0.03

### Progression forecasting metamodel evaluation

**Fig. 4:**
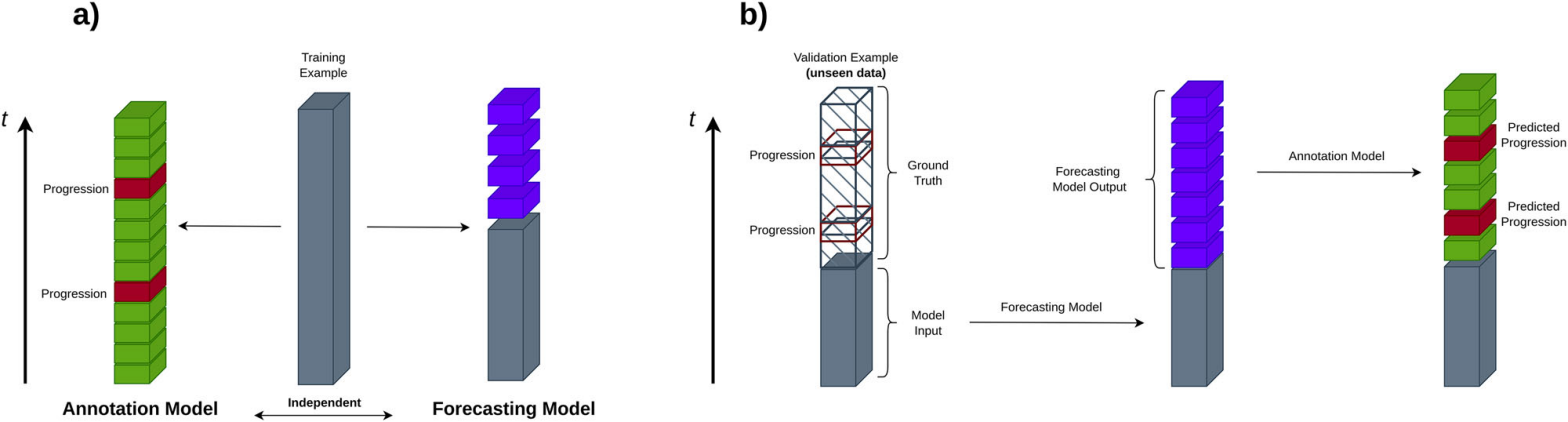
Interplay between forecasting and annotation models in progression event prediction. Model combination diagram illustrating the interplay between the forecasting and the annotation model. Both models were trained independently on the same training data (**a**) to perform their respective tasks. During validation (**b**), the forecasting model was given the first n follow-ups of a patient as input to forecast the next m follow-ups. The annotation model was then tasked to flag progression events within the forecasted data. The predicted progression events were evaluated against the actually observed future progression events in the downstream analyses.

**Fig. 5:**
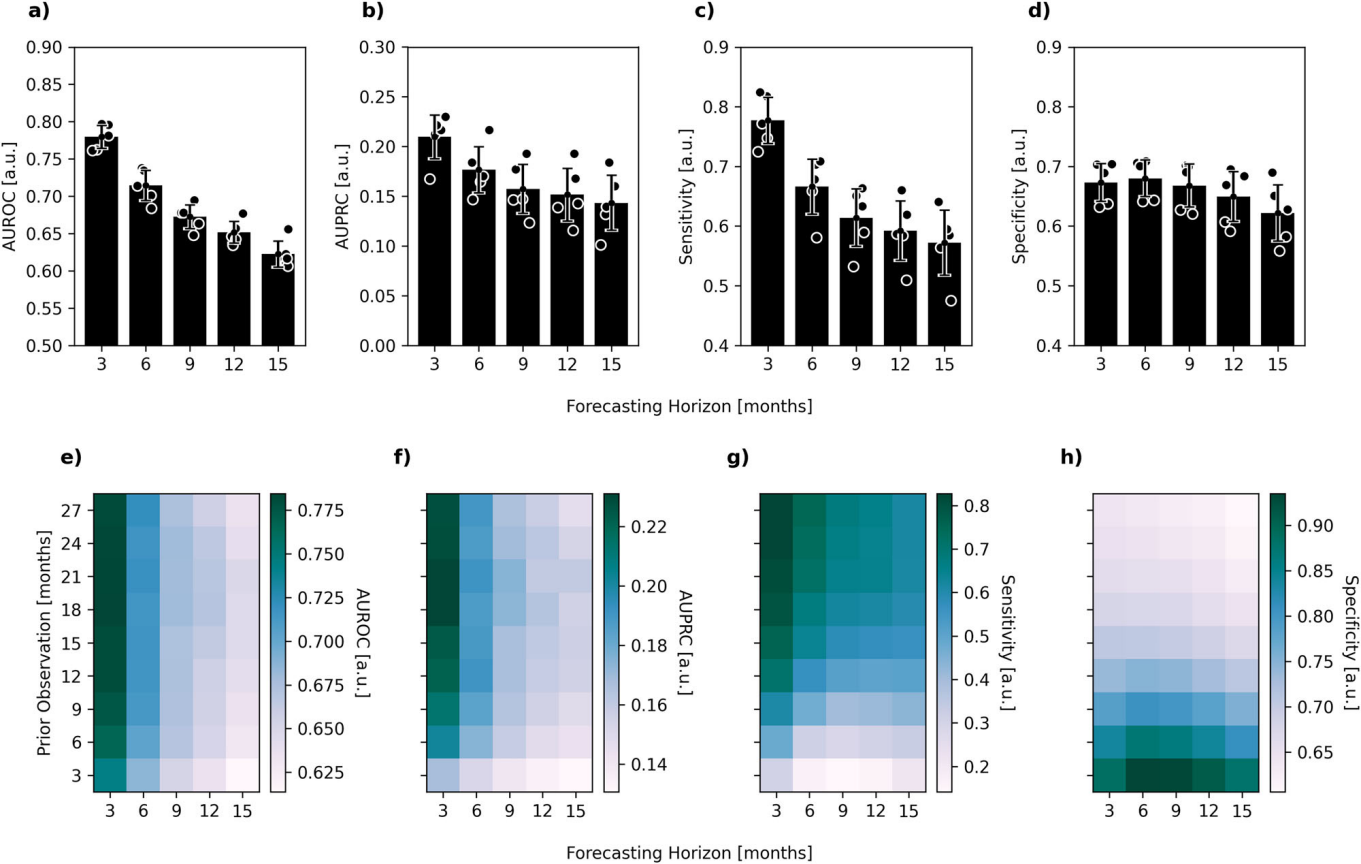
Comprehensive evaluation of forecasted progression events across multiple metrics. Evaluation of the forecasted progression events. The analysis includes overall metrics across all time points for five distinct forecasting horizons: 3, 6, 9, 12, and 15 months. Bars and error bars indicate mean ± standard deviation across the cross-validation folds. Metrics evaluated include (**a**) Area Under the Receiver Operating Characteristic curve (AUROC), (**b**) Area Under the Precision-Recall Curve (AUPRC), (**c**) Sensitivity, and (**d**) Specificity. Additionally, heatmaps display the AUROC (**e**), AUPRC (**f**), Sensitivity (**g**) and Specificity (**h**) charted according to the amount of available prior patient observation (*y*-axis) and forecasting horizon (*x*-axis). Values are reported as means across cross-validation folds.

**Fig. 6:**
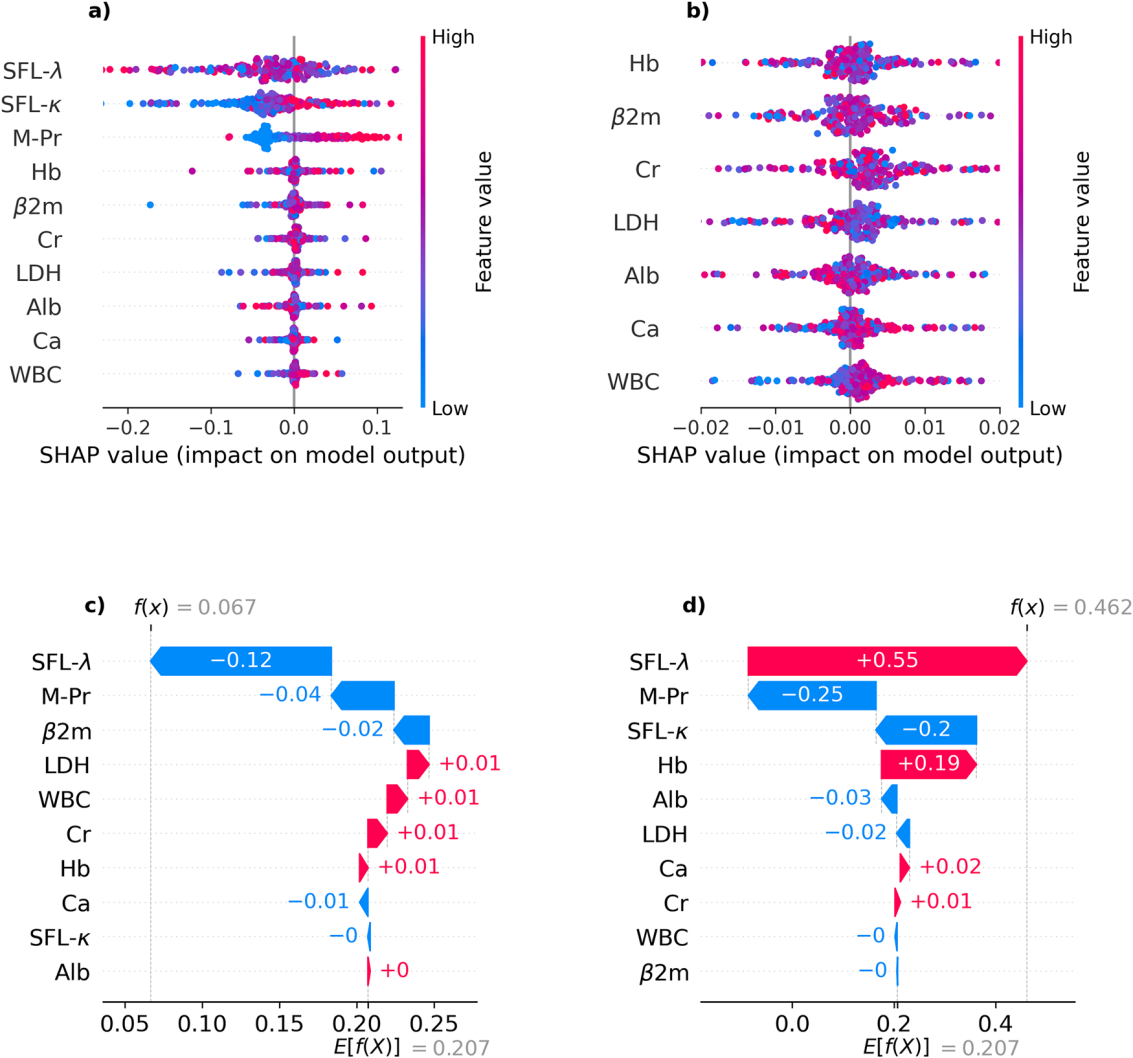
SHAP value analysis for metamodel predicting progression probability. SHAP values computed for the metamodel. The analysis includes (**a**) a beeswarm plot juxtaposing the effect of the ten input features’ values on the model output, which is the probability of progression. The x-axis indicates the magnitude and direction of the feature’s impact on the model’s output, while the color gradient reflects the feature values, with blue representing lower values and red representing higher values. **b** A detail cutout of panel a showing the more subtle effect of the bottom seven input features’ values on the model output. **c**, **d** Waterfall plots illustrating approximate explanations for the respective contribution of the input features in two instances where the model predicted a low (**c**) and a high (**d**) probability for progression at the next follow-up.

Finally, we integrated our annotation model with our forecasting model into a metamodel capable of predicting progression events by jointly forecasting blood work data and annotating the resulting trajectories (see methods sections Forecasting Model & Annotation Model). Figure 4 conveys an overview of the interaction of the forecasting model and the annotation model. We examined the combined models’ performance in predicting progression events depending on how far into the future the progression event would take place. In this scenario, the predictive power of the combination of both models started out high and showed a gradual decline as the forecasting horizon extended (Fig. 5a–d). As detailed in Fig. 5a, the combined models’ predictive performance, as measured by the AUROC, was 0.78 ± 0.02 at a 3 months forecasting horizon, diminishing to 0.62 ± 0.02 at 15 months. The AUPRC (Fig. 5b) followed a similar trend, starting at 0.21 ± 0.02 for 3 months and decreasing to 0.14 ± 0.03 for 15 months. Sensitivity (Fig. 5c) started at 0.78 ± 0.04 and declined to 0.57 ± 0.05, while specificity (Fig. 5d) slightly decreased from 0.67 ± 0.03 to 0.62 ± 0.05 over the same period. These findings suggest that our models can predict progression events with high accuracy for a 3 months forecasting horizon and, with reliability that is still significantly beyond chance even for forecasting horizons extending beyond 12 months. An additional layer of analysis was conducted to determine the impact of the amount of available prior patient observation on the accuracy of future forecasts. Interestingly, the length of prior observation had little effect on short-term forecasting but became increasingly important for longer forecasting horizons. This was visually represented by a color gradient in the heatmaps (Fig. 5e, h), with decreasing intensity from the upper left corner (long prior observation and short forecasting horizon) to the lower right corner (short prior observation and long forecasting horizon). This trend was consistent across the AUROC, AUPRC and Sensitivity (Fig. 5e-g). For the AUROC heatmap (Fig. 5e), values are largely uniform across prior observation periods, with a slight tendency towards lower values at short prior observation periods. Notably, the AUROC values fall within a range of approximately 0.62 to 0.78. The AUPRC heatmap (Fig. 5f) appears to have a slightly more pronounced tendency to increase with larger prior observation periods. The values are centered around a range of approximately 0.13 to 0.23. Sensitivity (Fig. 5g) presents the highest variance across different observation periods and forecasting horizons. The values range from around 0.18 to 0.82, with a visually discernible patterning that suggests an increase in sensitivity associated with longer prior observation periods, indicating a stronger ability of the model to correctly identify progression events when provided with more historical data, especially when forecasting nearer-term events. Lastly, the heatmap corresponding to specificity (Fig. 5h) shows a broad spectrum of values as well, varying between approximately 0.63 to 0.92. A counter-intuitive pattern emerged when examining the specificity, which was highest for short prior observations and longer forecasting horizons. We saw that in scenarios, where there is little prior information available, the forecasting model engages in a quasi-random walk, devoid of meaningful information, leading the annotation model to predominantly label time points as non-PD. This tendency resulted in an artificially inflated specificity, rather than predictive performance of the model. In summary, these heatmaps deliver a comprehensive overview of the model’s performance, highlighting that while AUROC and AUPRC remain relatively stable, Sensitivity and Specificity appear to be more sensitive to the duration of prior observation and the extent of the forecasting horizon. This suggests that the length of prior observation, in conjunction with the forecasting horizon, plays a critical role in the model’s ability to correctly predict impeding progression events. Looking at individual patient-level predictions, in Supplementary Fig. 3 we present an analogous analysis to the one presented in Fig. 2, where we demonstrate the prediction of our Progression Forecasting Metamodel for a single patients. Here, we highlight instances where the model correctly predicted future progression as well as demonstrating where it did not. Notably, in the cases presented in Supplementary Fig. 3a and Supplementary Fig. 3b, disease progression is not immediately apparent from any single clinical marker. However, the predictive model correctly flagged these timepoints (red shaded areas). In contrast, the case presented in Supplementary Fig. 3c shows several progression events that are readily identifiable from the trajectories of single disease markers, yet the model failed to flag them (hatched areas). These examples illustrate the model’s ability to capture the multifactorial nature of disease progression in multiple myeloma, while also underscoring the need for cautious interpretation of its outputs. Intrigued to elucidate the metamodel’s mechanism for integrating the disease markers for predictions, we again conducted a SHAP value analysis (see methods section SHAP Value Computation). Figure 6 provides a detailed analysis of the approximate contributions of individual features to the metamodel’s predictions for future disease progression. Panels (a, b) display summary plots of SHAP values for all features, where each dot represents a single prediction. Notably, the key disease parameters SFL-λ, SFL-κ and M-Pr exhibit strong SHAP values (Fig. 6a), suggesting their significant influence on the model’s predictions. In contrast, panel (b) highlights features with more subtle contributions, such as Alb and WBC (Fig. 6b). Here, a slight tendency for high levels of WBC and low levels of Alb to drive an increase in progression probability is displayed. This observation aligns with clinical expectations as inflammation and Hypoalbuminemia are well-established indicators of poor prognosis in MM. Figure 6c, d present SHAP waterfall plots for specific prediction instances, illustrating how individual features cumulatively influence the model’s output. In panel (c), SFL-λ contributes a large negative SHAP value, driving the prediction below the expected prior, with M-Pr and β2m also playing non-negligible roles in lowering the prediction. Conversely, in panel (d), SFL-λ strongly pushes the prediction upwards, complemented by a positive contribution from Hb, which counteracts negative effects from M-Pr and SFL-κ. These waterfall plots offer granular insight into how the biomarkers drive individual predictions. Taken together, the SHAP value analysis demonstrates the model’s ability to capture the varying influences of biomarkers, highlighting the critical roles of M-Pr, SFL-λ and SFL-κ in driving predictions while also accounting for the more subtle effects of other features.

### External validation on GMMG-MM5 study dataset

**Fig. 7:**
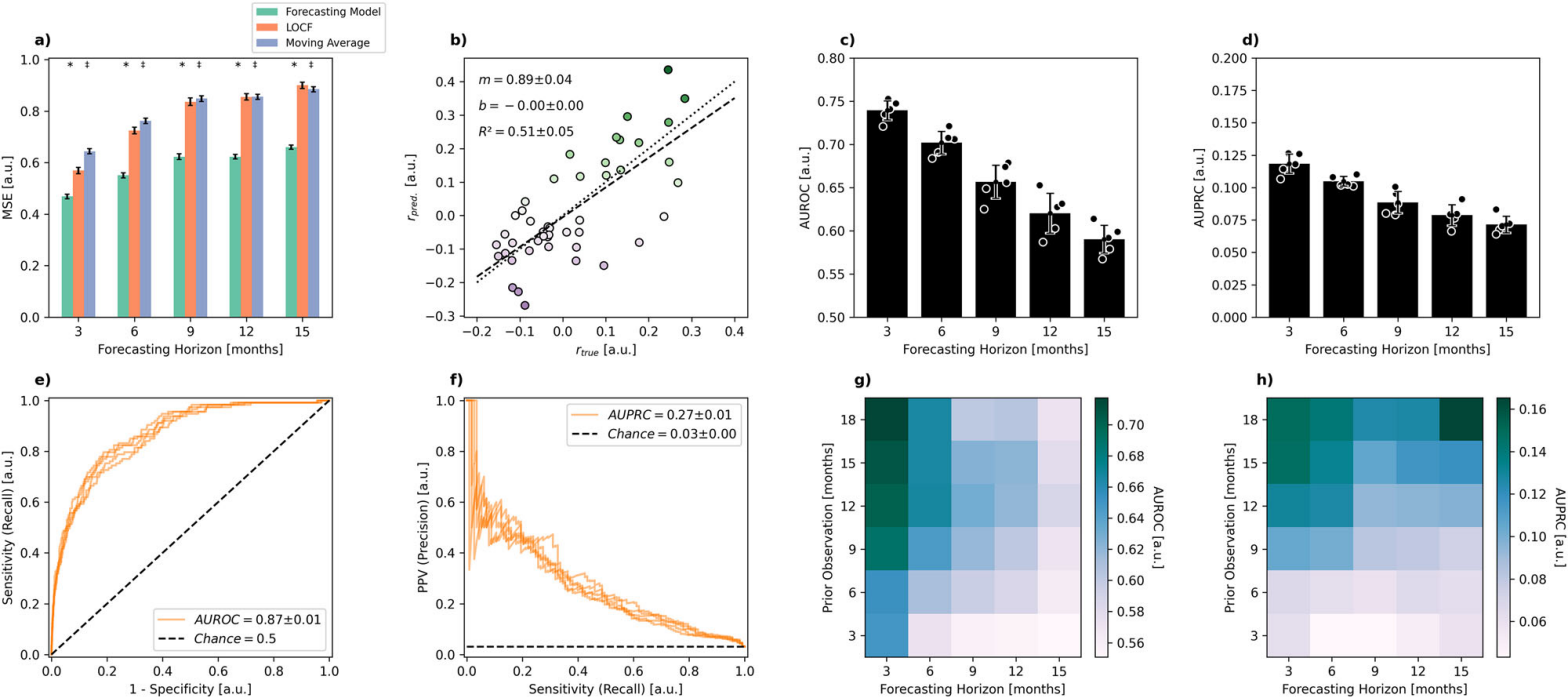
External validation of models on the GMMG-MM5 study dataset. External validation of the models on the GMMG-MM5 study dataset. The analysis includes (**a**) aggregated mean squared error (MSE) of the forecasting model compared to the baseline methods last observation carried forward (LOCF) and moving average (MA) across varying forecasting horizons (3, 6, 9, 12, and 15 months). Cross and asterisk symbols (comparison of the forecasting model and LOCF) and dagger and double dagger symbols (comparison of the forecasting model and MA) indicate *p* < 0.1 and *p* < 0.05 respectively. **b** Juxtaposition of the correlation coefficients from the actual data with those obtained from the forecasted data across varying forecasting horizons. Dashed lines show the lines of best fit. Parameters are reported as mean ± standard deviation across cross-validation folds. Dotted lines show the expected lines of best fit for m = 1 and b = 0. **c**, **d** Discriminative performance expressed as area under the receiver operating characteristic curve (AUROC) (**c**) and area under the precision-recall curve (AUPRC) (**d**) of the combined model at predicting progression events across varying forecasting horizons. Bars and error bars indicate mean ± standard deviation across the cross-validation folds. **e**, **f** Discriminative performance of the annotation model at baseline. **e** ROC curve, with an AUROC of 0.87 ± 0.01. **f** PRC, with an AUPRC of 0.27 ± 0.01, significantly exceeding chance (0.03 ± 0.00). **g**, **h** Heatmaps of the combined model’s discriminative performance expressed as AUROC (**g**) and AUPRC (**h**) as a function of the prior observation period and forecasting horizon.

To validate the generalizability and robustness of our prediction metamodel, we applied it to the GMMG-MM5 study dataset (*N* = 504), which was conducted by the German-Speaking Myeloma Multicenter Group (GMMG). We identified the GMMG-MM5 dataset as suitable for external validation due to largely similar patient demographics, treatment regimes and monitoring intervals, and hence repeated our core analyses (Fig. 7). The results reproduced the main findings of the study, demonstrating that the model consistently outperforms baseline estimators in forecasting biomarker trajectories across all time horizons with high significance (*p* < 0.05; Fig. 7a), confirming its superior accuracy and adaptability to unseen data. Detailed performance analyses at baseline (panels e and f) show that the model achieves an AUROC of 0.87 ± 0.01 and an AUPRC of 0.27 ± 0.01, far exceeding the chance level (0.03 ± 0.00). Importantly, the AUPRC values indicate that the model retains its ability to identify progression events, even in the context of an imbalanced external dataset where progression cases are relatively rare (3% of all time points). The model’s discriminative performance was assessed using AUROC and AUPRC metrics as a function of forecasting horizon (panels c and d). As observed previously, the model demonstrates high discriminative power at shorter forecasting horizons, with AUROC and AUPRC values decreasing as the horizon lengthens. These metrics underscore the models’ clinical utility in identifying patients at immediate risk of progression, particularly in the critical short-term window where early intervention is most impactful. Finally, the interaction between the length of prior observation periods and forecasting horizons (Figures g and h) reveals a pattern that is consistent with internal validation. As with the primary dataset, performance improves with longer observation windows, especially at shorter horizons. This finding highlights the model’s ability to leverage longitudinal biomarker data for accurate predictions. However, the diminishing gains in performance for longer horizons reinforce the notion that recent biomarker dynamics are more predictive of near-term outcomes. Taken together, these results demonstrate the robustness of the forecasting model when applied to independent, external data. By reproducing the main findings of the study, the external validation confirms that the model’s predictions are generalizable and clinically relevant. This enhances confidence in the model’s potential utility for broader clinical applications, including risk stratification and personalized follow-up strategies in diverse patient populations.

## Discussion

In this study, we demonstrated how risk of impeding disease progression for individual MM patients can be inferred from the kinetics in the unique trajectories of their routine blood work data. For patients with MM, the ability to anticipate future progression events is integral to effective patient care as this foresight allows for individualized risk assessment and the opportunity to adjust follow-up schedules and treatment plans proactively. To this end, we developed a modular prediction model adept at generating forecasted blood work data and flagging progression events within them. The robustness of our model was assessed using a 5-fold cross-validation to evaluate the model’s performance across different subsets of the data. The results of the cross-validation process are encouraging. The models demonstrated a remarkable consistency in performance across all five folds, as evidenced by the very low variance in the relevant metrics. Such consistency suggests that the models are not overly sensitive to specific partitioning of the data and can generalize well to new data. A major strength of using the Multiple Myeloma Research Foundation (MMRF) CoMMpass dataset to generate our virtual twin model is the wide range of state-of-the-art treatments used in the study as well as the heterogeneous patient population ranging from fit individuals, who are eligible for transplant, to frail patients treated with conventional therapies.

To further strengthen the validity of our study, we utilized the GMMG-MM5 study dataset to externally validate our models ensuring a robust test of their predictive power in a truly independent context. This allowed us to assess the models’ resilience to differences in data collection methods, demographic variations, and other external factors that could affect their performance. Because our model proved to be efficacious irrespective of the type of therapy and patients status and showed close to equal performance when applied to an independend dataset, we are confident about the reproducibility in a wide range of clinical settings. Additionally, exploring the potential of targeting a subset of patients with more predictable laboratory value trends could further enhance our model’s performance and provide a safer context for assessing its clinical utility in future work. However, one limitation that should be noted is that our model’s current annotation of progression events exhibits a high rate of false positives as evident by the AUPRC. Here, it is important to contextualize this finding within the broader landscape of diagnostic testing in low-prevalence settings. In scenarios where the actual incidence of a condition is rare, even tests with very high accuracy yield a large proportion of false positives. This phenomenon is a consequence of the PPV being inherently dependent on the prevalence of the condition in question^23,24^. This is a well-documented challenge in medical diagnostics and affects a wide range of screening and monitoring tools^24–26^. The clinical utility of a predictive model is thus not solely determined by its precision but also by the balance between the costs and benefits of false positives and false negatives. Based on this, we propose that our model be used to complement clinical judement by identifying at-risk patients early in a data-driven manner, thereby facilitating timely follow-up appointments, rather than recourse to invasive diagnostics or treatments. This approach allows for more frequent monitoring, potentially improving patient outcomes through earlier intervention. According to the performance metrics at the 3 months forecasting interval illustrated in Fig. 3c and Fig. 5a (Sensitivity = 78%, Specificity = 66%, Prevalence = 7%), implementing our model in a clinical setting to alert for risk of progression within the immediate follow-up interval would lead to earlier follow-up for approximately 36% of all patients, with a PPV amounting to 15%. This is notably higher than the PPVs reported for many screening methods across various types of cancers, which typically fall below 10%^27–30^, offering a substantial improvement over other screening protocols. In external validation, our model demonstrated a Sensitivity of 72% and Specificity of 59% for predicting progression within the immediate 3 month interval. It is important to note that the prevalence of progression in the GMMG-MM5 cohort was significantly lower, at 3% (Fig. 7f), compared to 7% (Fig. 3c) in the CoMMpass cohort, which is characteristic of a study dataset rather than a real-world clinical dataset. As outlined above, while prevalence does not influence the Sensitivity and Specificity of a diagnostic test, its PPV inherently depends on it. Therefore, a fair comparison of test performance across datasets necessitates euqal prevalence assumptions. When assuming a progression prevalence of 7%, the model’s PPV in the external validation yields approximately 11.7%, which still exceeds the PPVs commonly reported for many cancer screenings. This underscores the potential of our model to significantly enhance early detection and management of disease progression promising to prevent biochemical progressions from evolving into clinical progressions, which are associated with worse patient outcomes^31^. Moreover, the deployment of such a model could be justified in settings where the cost of missing an actual progression event is substantially higher than the inconvenience or cost of additional follow-up. The decision to implement such a model should be informed by a thorough cost-benefit analysis, considering the specific clinical context and the available resources. The models’ high sensitivity when extensive historical patient data is available could be particularly beneficial in early detection and monitoring scenarios, provided that the healthcare system can accommodate the resultant need for increased follow-up. Refinement of the model, particularly in conjunction with other diagnostic tools, could enhance its precision and expand its applicability in clinical practice even further. Additionally, while our methods effectively quantify uncertainty within the confines of available data and predefined model structures, they do not fully account for systemic uncertainties that might arise from external factors such as changes in treatment protocols or new diagnostic criteria. Our validation metrics, primarily AUROC and AUPRC, while robust, do not capture all aspects of clinical utility, such as the cost-effectiveness of interventions based on model predictions or the impact of false positives on patient outcomes. Furthermore, the dynamic nature of MM and its treatment landscape means that the digital twin must continuously evolve, posing challenges for maintaining sustained model accuracy and relevance over time. These limitations underscore the need for ongoing Verification Validation and Uncertainty Quantification (VVUQ) processes and the exploration of more adaptive modeling techniques that can better accommodate the complexities and evolving nature of clinical environments. The selection of an appropriate model for time series forecasting is pivotal to the success of any predictive analysis. In this study, we opted to utilize a LSTM network over traditional methods such as AutoRegressive Integrated Moving Average (ARIMA) models. This decision was informed by several factors that align with the unique characteristics of our data and the requirements of our predictive tasks. Comparative studies have consistently shown that LSTMs outperform traditional methods, especially in scenarios where the data exhibit complex temporal dependencies^32–34^. The inherent structure of our data is multivariate, with several covariates influencing the target variables. LSTMs are intrinsically designed to handle multivariate time series data, allowing them to capture the interdependencies among different covariates effectively^8^ rather than considering each covariate’s trajectory in isolation. This is a significant advantage over ARIMA and similar methods, which typically cannot natively accommodate multivariate time series^32^. One of the most compelling reasons for choosing LSTMs over traditional methods is their feature learning capability. This attribute eliminates the need for manual feature engineering, which can be time-consuming and may not capture all the predictive signals present in the data. This allows LSTMs to learn from and adapt to the intricacies of the data, leading to more robust and generalizable models. In interpreting the results of our SHAP value analysis, it is essential to consider the specific characteristics of the LSTM architecture employed in our models. Notably, LSTM models inherently account for temporal dependencies in data, capturing dynamic changes over time that significantly influence predictions. For the purposes of this analysis, we simplified our approach by examining interactions at single time points, thus not incorporating the temporal dimension. This methodological choice was made to clarify the direct influence of individual features on our models’ predictions. However, it is important to acknowledge that a more comprehensive analysis that includes temporal dynamics could potentially reveal additional insights into how feature variations over time impact predictions, which at present our analyses do not encompass. Furthermore, our forecasting model incorporates a CRBM, which inherently produces probabilistic outputs rather than deterministic point estimates. Consequently, the true SHAP values derived from our models are not single values but distributions that reflect the uncertainty inherent in the models’ predictions. In our analysis, we presented these SHAP values as their expected values to maintain clarity and interpretability. This reduction, while useful for visualization and discussion, omits the probabilistic nature of the models, which could provide deeper insights into the variability and reliability of feature contributions. Lastly, while the SHAP value analysis provides valuable insights into the relationships between features and their impact on model predictions, as illustrated in Supplementary Fig. 4, it is crucial to interpret these findings within the context of the model’s complexity. The interactions depicted do not necessarily imply direct biological causations but rather stochastic associations learned by the model within the training dataset’s constraints. While the correspondences with known biological relationships lend credibility to the model’s utility, they should be viewed as part of a broader, complex interplay of features rather than definitive causal relationships. For example, in the case of patient *MMRF_1569* shown in Fig. 2c, the model predicted a decrease in β2m despite stable historical levels. While this may seem counterintuitive, it is important to consider that the model’s predictions are based on the integrated effects of all parameters, not just the historical trajectory of a single marker. Here, the model’s prediction may have been influenced by changes in other parameters or their interactions, which are not immediately apparent. However, we also acknowledge that there are epistemic factors — such as clinical influences — that our model does not account for. These factors can affect the trajectories of parameters like β2m and may lead to predictions that do not fully align with subsequent observed values. These considerations underscore the sophistication of our predictive model and highlight the importance of a nuanced approach to interpreting machine learning analyses in biomedical research. This is especially highlighted by the results presented in Supplementary Fig. 3. These examples emphasize the promise of predictive models in integrating multiple disease markers to overcome challenges associated with the interpretation of isolated markers, while also underscoring importance of exercising caution when taking their outputs at face value. The ability to accurately forecast blood work holds significant clinical value, as it provides a window into the patient’s future health status, allowing for preemptive medical interventions. We have designed our prediction system as a modular pipeline, which features distinct entities for forecasting future blood work and for annotating progression events within these forecasts. This modular approach facilitates interoperability and promises the possibility of integrating further modules for the annotation of alternative endpoints within the same system. For instance, anticipating a drop in hemoglobin levels could prompt early iron supplementation or transfusions. Similarly, forecasting a decline in white blood cell counts might lead to prophylactic measures to reduce the risk of infection before leukopenia sets in. While disease progression was the primary endpoint we focused on in this study due to its clinical significance, the predictive modeling of blood work holds the potential to serve as a tool to anticipate a broader range of clinical endpoints, which would help guiding patient maintenance and thus enhance the quality of care. This will be the scope of our future research endeavors. Moreover, our approach utilizes a minimal set of routine blood work measurements, avoiding the need for expensive or labor-intensive tests. Although this design choice enhances practicality and broadens accessibility in clinical settings, a comprehensive patient model should ideally incorporate the genetic profile of the disease, including cytogenetic abnormalities. Furthermore, our model currently does not include real-time clinical factors such as patient symptoms, treatment side effects, or imaging profiles, which play a vital role in clinical decision-making. Because of this, it is important to note that at present our tool should be considered as reaserch-use-only and is not (and should not be) intended to replace clinical judgment. Instead, it is intended to complement clinical judgment by providing data-driven insights alongside comprehensive patient assessments. While our model currently does not include these features, we plan to expand our system to integrate them in our future work. Given the beforementioned limitations, we want to emphasize the importance of careful and considered implementation of ML in healthcare settings an reinforce that they should be used to complement clinicians’ throught processes rather than narrowing them. Nevertheless, our model demonstrates a paradigm shift from classical prognostic risk evaluation based on initial assessments towards a framework in which a prediction of risk is made for specific future time points. Moreover, the predictions of the models can be verified upon follow-up of the patient and its predictive capability can be periodically refined by updating predictions as soon as new patient data becomes available. This interaction between patients and their computational representation allows to derive more informed treatment and health care decisions by e.g. shortening follow-up intervals for more close monitoring or early intervention at impeding risk of disease progression. This foresight can be pivotal in clinical decision-making, enabling healthcare providers to implement timely therapeutic strategies to mitigate or prevent the adverse effects associated with such hematologic conditions. In summary, our proposed system addresses several key challenges identified for the adoption of digital twins in healthcare^19^:

- We exemplify the development of advanced, patient-specific predictive models (B1).
- By relying on routine blood work data, we address the need for representative, available data for model development and validation (B2).
- Our modular approach enhances interpretability and facilitates integration with additional modules, supporting the VHT’s emphasis on interoperability and scalability (B5).

By addressing these challenges, our system contributes to the realization of a scalable and cost-effective VHT ecosystem in MM care, which could help improve patient outcomes and optimized healthcare resource utilization in MM.

## Methods

### Ethics statement

The CoMMpass study was funded by the Multiple Myeloma Research Foundation (MMRF) and conducted in line with the Declaration of Helsinki. Approval for the study was granted to the MMRF by the second panel of the Western Institutional Review Board. The CoMMpass study data is publicly deposited by the MMRF in anonymized form (refer to section Data availability). Participants were not paid for their involvement in the CoMMpass study and only joined the study after giving their written informed consent. The MM5 trial was carried out in compliance with the European Clinical Trial Directive (2005) and the Declaration of Helsinki. The GMMG received ethical approval from the University of Heidelberg’s ethics committee. Participants were not paid for their involvement in the MM5 study and only joined the study after giving their written informed consent. The MM5 study data was shared with the University of Leipzig in anonymized form and was processed in a GDPR-compliant manner. All research in this study was carried out retrospectively and in compliance with the Declaration of Helsinki.

### Training and internal validation patient characteristics

For training and internal validation, data from patients with NDMM were retrieved from the CoMMpass study (NCT01454297) database version IA21. To model MM disease kinetics, we selected the following parameters for our predictive framework due to their respective importance in MM disease monitoring:

- Hb as anemia is a common complication in MM patients^35,36^.
- Ca due to its importance for bone turnover and hypercalcemia, which 30% of MM patients present with^37,38^.
- Cr due to its involvement in the highly prevalent renal insufficiency of MM patients^39,40^
- WBC counts, since leukopenia is a common side effect of anti-myeloma therapies and directly connected to infectious complications during therapy^41–44^.
- M-Pr, SFL-κ and SFL-λ as MM specific markers for disease activity^37^
- LDH, Alb and β2m due to their prognostic value for survival outcomes^3^.

Furthermore, we have chosen the above parameters because they constitute routine measurements in MM care and do not require costly or labor-intensive test and are therefore easily accessible in routine clinical practice if not already available. Missing values were imputed through LOCF. If no value was available for LOCF, we would carry the earliest available measurement backward. Our rationale was to prevent cross-patient imputations and to mirror clinical practice as closely as possible. The only patients excluded were those for which any of the before mentioned parameters were never measured across all their visits. A total of 875 patients remained with an average of 19 ± 9 (mean ± s.d.) visits per patient. We split this group into five distinct sets of 175 patients each for cross-validation purposes. Thus, in each of the five folds, there were a total of 700 patients with 13,420 ± 87 unique time points available for training and 175 patients with 3355 ± 87 unique time points for validation. Out of these, 989 ± 30 time points marked progression events for training and 247 ± 30 for validation. In Table 2 the relevant patient characteristics and distribution of data across the five folds are displayed.

**Table 2:** Patient characteristics table separated by cross-validation (*k*^*th*^) fold

k^th^-fold	k =1	k = 2	k = 3	k = 4	k = 5
Patients	175	175	175	175	175
Female	73	55	79	67	79
Male	102	120	96	108	96
Age	65 ± 11	64 ± 10	64 ± 10	62 ± 10	63 ± 11
Stem Cell Transplantees	97	108	87	99	103
**ISS**					
1	56	69	53	63	58
2	70	56	61	62	64
3	46	47	59	48	51
**Treatment classification**					
Bortezomib-based	39	33	42	33	40
Carfilzomib-based	2	0	2	0	0
IMIDs-based	11	14	6	8	10
combined IMIDs/carfilzomib-based	11	11	9	12	8
combined bortezomib/IMIDs-based	107	104	111	112	107
combined bortezomib/IMIDs/carfilzomib-based	5	12	5	9	9
combined bortezomib/carfilzomib-based	0	1	0	0	1
combined daratumumab/IMIDs/carfilzomib-based	0	0	0	1	0
Total Visits	3435	3269	3281	3483	3307
Total Progression Events	240	253	197	255	292
**Lab value counts**					
Hemoglobin	3056	2902	2806	3112	2876
Calcium	2992	2847	2748	3043	2799
Creatinine	3017	2863	2778	3066	2822
LDH	1999	2006	1882	1913	1951
Albumin	2929	2784	2711	3009	2756
β-2-Microglobulin	1061	1028	850	791	977
M-Protein	2622	2585	2379	2692	2447
SFL-λ	2806	2605	2503	2826	2630
SFL-κ	2818	2612	2510	2834	2625
WBC	3057	2904	2802	3108	2877

### External validation patient characteristics

For external validation of our model, we used data from the GMMG-MM5 phase III trial (EudraCT No. 2010-019173-16)^45–47^, conducted by the GMMG. For a comprehensive description of patient and disease characteristics, as well as the study design, we refer the reader to the original publication by Mai et al.^45^. In brief, in 504 patients bortezomib/cyclophosphamide/dexamethasone (VCD) was compared to bortezomib/doxorubicin/dexamethasone (PAd) with an average of 10 ± 4 (mean ± s.d.) follow-ups per patient.

### Data preprocessing

All blood work parameters used in this study were subject to a feature-wise power transformation using the PowerTransformer class of scikit-learn^48^ (version 1.4.1.post1) to approximate a Gaussian-like distribution of the data. The PowerTransformer was fitted exclusively on the respective training portions of the data in each of the five cross-validation folds (80%) and only applied to the validation portions (20%) for transformation. Supplementary Fig. 5 contains QQ-plots of the distributions of the training and validation data after power transformation. For external validation, each of the pre-fitted PowerTransformers were applied to the entirety of the GMMG-MM5 dataset for transformation. Likewise, the pre-trained models from the five cross-validation folds were applied to the entirety of the GMMG-MM5, allowing for a comparison of models between folds in an external validation setting.

### Long short-term memory conditional restricted Boltzmann machine

Disease trajectories in MM present a unique challenge due to their high heterogeneity and the presence of temporal patterns that operate over variable time scales^21^. The predictive modeling of disease trajectories in MM requires the analysis of these temporal patterns to understand how they evolve over time. Recognizing these complexities, we have implemented a hybrid model that utilizes a LSTM network in conjunction with a Conditional Restricted Boltzmann Machine (CRBM), a class of generative stochastic artificial neural networks^49^. CRBMs are a variant of the Restricted Boltzmann Machine^50^, which extend their capabilities by incorporating conditional dependencies on external or previous information. In the context of disease trajectory modeling, this allows the model to learn the distribution of data conditioned on past information, making it particularly suitable for time-series data where the future state is dependent on observed historical data. Because the outputs of CRBMs are inherently probabilistic, they facilitate distributions of potential disease trajectories, rather than yielding a single, deterministic forecast devoid of any quantification of uncertainty. Such a probabilistic approach is particularly advantageous in medical applications where the exact future cannot be determined. Hence, CRBMs were shown to be effective in modeling trajectories of diseases such as in Alzheimer’s^51^ and multiple sclerosis^52^. However, the complexity of temporal dependencies that CRBMs can capture hinges on the number of previous time-lagged steps a prediction is conditioned on – a design choice made when implementing the model^51^. LSTMs address this challenge with their architecture: They are composed of modules with gating mechanisms – the input, output, and forget gates – that regulate information flow. This allows the network to learn to retain relevant information and to discard the non-essential^8^. This selective memory enables the model to adaptively focus on the most predictive features over time. Subsequently, the CRBM takes on the role of generating future time steps, conditioned on the internal state of the LSTM. This approach ensures that predictions are always anchored to the full spectrum of available observations, rather than being limited to the most recent ones. In summary, our design choice was motivated by the following rationale:

1. The LSTM serves as a tool to encode the entire history of a patient’s sequential blood work, effectively summarizing all prior observations within its internal state. This encoding is crucial for capturing the multi-scaled temporal patterns inherent in MM.
2. The integration of the CRBM facilitates a paradigm shift from traditional regression-based forecasting to probabilistic modeling. By generating probabilities conditioned on the encoded patient history from the LSTM, the CRBM provides a spectrum of possible outcomes rather than a single point prediction, thereby offering a more comprehensive understanding of uncertainty in MM disease trajectories.

**Fig. 8:**
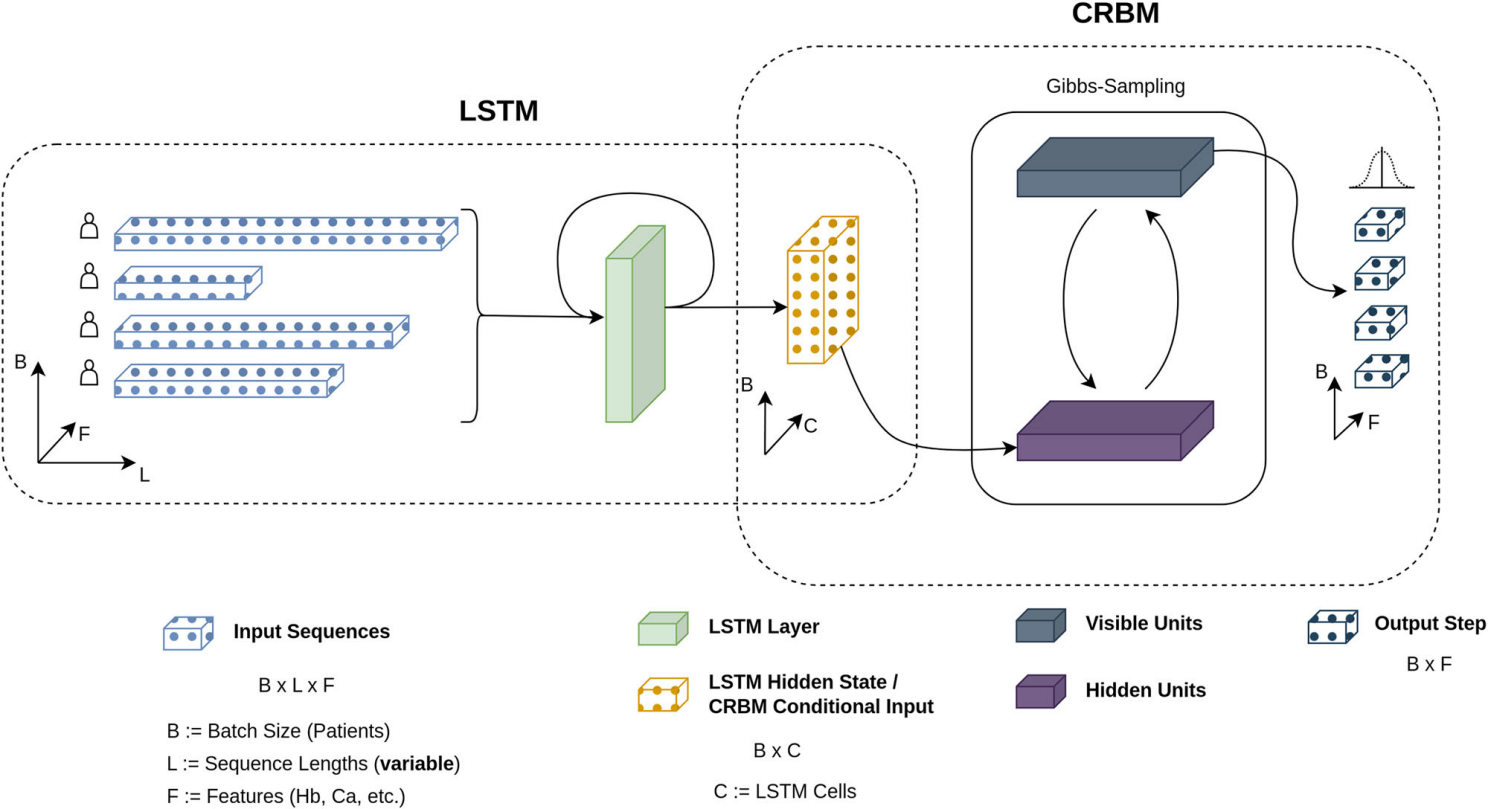
Architecture diagram of the forecasting model. Architecture diagram of the forecasting model. The Long Short-Term Memory (LSTM) network summarizes all observations of a patient’s sequential blood work within its internal state. The Conditional Restricted Boltzmann Machine (CRBM) then generates probabilities of possible outcomes, conditioned on the patient history encoded by the LSTM, through a Gibbs-sampling algorithm, yielding a distribution over possible future states for the input variables. Model outputs can be fed back into the LSTM for recurrent generation of future observations.

Our hybrid model, therefore, offers a more contextually aware framework to address the complex and variable temporal dynamics of MM. Figure 8 provides an overview of the model architecture and its internal information flow. The LSTM-CRBM hybrid model is referred to as the Forecasting Model.

### Forecasting model

The LSTM component of the forecasting model was configured with 10 input features which we selected as described in Training and Internal Validation Patient Characteristics and consisted of 32 memory cells. The CRBM component was greatly inspired by the Neural Boltzmann Machine architecture proposed by Lang et al.^53^, with a significant adaptation that simplified the complexity of the model; We removed the latent spaces in the Bias-, Precision-, and Weights-networks, which decreased its computational complexity and potential for overfitting. The CRBM was subsequently structured with 32 features in its input layer and 16 hidden units, which are connected to 10 visible units. Total parameters: 11,572. Blood work was forecasted according to methods section Monte Carlo Simulation.

### Annotation model

Given the critical importance of timely recognition of progression events in the management of MM, we were motivated to develop an annotation model able to identify and flag such events within blood work time series data. We pursued the development of this model with the goal of applying it to the forecasted blood work data to identify progression events well before they would otherwise become apparent. The deployment of the model served two pivotal functions:

1. Mitigating biases inherent in manual annotations and
2. addressing the absence of certain features in routine tests, which are typically employed to annotate remission criteria according to the IMWG^21^.

To annotate a specific time point, the time series comprising all previous observations up to and including the current one were used as input for the annotation model. Based on this, the model was trained to label the current time point as either PD or non-PD. Labels were inferred from treatment response documentation in the CoMMpass dataset. We observed that the prevalence of progression events across all time points in the data set was 7% ± 1%. Therefore, to ensure a robust training process, we balanced the dataset by upsampling the PD time series instances, ensuring the model was trained on an equal representation of both categories. Testing was always done on the representative, imbalanced data. The annotation model only utilized M-Pr, SFL-κ, SFL-κ, as well as the SFL ratio and the SFL differences as input features. Based on this, the Annotation Model employs an LSTM layer with 6 input features and 8 memory cells. Following the LSTM, a fully connected dense layer with 8 input features and 2 output features is applied. The dense layer utilizes a softmax activation function to output a probability distribution over the two annotation labels. Total parameters: 530.

### Model implementation and training

All employed models were implemented using the PyTorch^54^ framework (version 2.2.0) and trained using PyTorchLightning^55^ (version 1.9.5). The Forecasting model was trained in 100 Epochs with a batch size of 32 using the following weight decay parameters for the individual components:

- LSTM: 0.1
- Precision-Net: 0.1
- Bias-Net: 0.1
- Weights-Net: 0.2

The Annotation model was trained in 200 Epochs with a batch size of 128 using the following weight decay parameters for its components:

- LSTM: 1
- Dense: 1

Model hyperparameters and training parameters were determined through grid search. The forecasting model was trained with *Contrastive Divergence* loss and the annotation model was trained with *Binary Cross-Entropy* loss. Both models were trained with a learning rate of 0.0001 using the AdamW optimizer^56^.

### Monte Carlo simulation

The CRBM component of our forecasting model generates predictions by virtue of a Gibbs-sampling algorithm. For an in depth explanation of the sampling algorithm, we would like to refer the reader to the paper by Lang et al.^53^ about the original implementation of a Neural Boltzmann Machine which we adopted for our model as described in methods section Forecasting Model. In brief, to generate a prediction for a given time point, we drew 1,000 samples employing 32 Markov-Chain steps per sample using all previous observations leading up to the one in question as input for our model. We obtained the most probable estimate for each respective time point by averaging the resulting distribution of samples. For multi-step predictions, we were drawing several samples in a recurrent loop, i.e. sample the immediate next step, reinserting it into the model, and then sampling successively until the desired forecasting horizon was reached. This process enabled us to generate distributions of complete trajectories rather than isolated time points. In coherence with single-step predictions, 1,000 trajectories were sampled and averaged to yield the most probable trajectory for a given patient.

### Verification, validation and uncertainty quantification

In accordance with best practices for VVUQ of digital twin systems, as outlined in the National ResearchCouncil’s report *Assessing the Reliability of Complex Models*^57^, our methodology addresses the components of VVUQ as follows; Verification was conducted by addressing to which degree the forecasting model’s predictions replicated actual patient data, ensuring that the model accurately forecasts blood work values and effectively reflects changes between sequential measurements and maintains the integrity of cross-correlations between features and their lagged autocorrelation. To this end, we quantified the MSE between the predicted and actual blood work values for our model and compared it with trivial predictive benchmarks using LOCF and MA. We further utilized linear regression to obtain the slope, intercept and coefficient of determination (R^2^) to assess the accuracy of the predicted cross-correlations between features, as well as Pearson’s correlation coefficient (*r*) to assess the correlation of changes in the individual features in the forecasted blood work and actual patient data. Validation was achieved through statistical measures; the AUROC and the AUPRC were utilized to assess the discrimination between progression and non-progression time points and to evaluate the predictiveness of positive labels, respectively. The combined efficacy of the forecasting- and annotation model was further validated by calibrating an optimal decision boundary and calculating the resulting sensitivity and specificity of the models’ labeling. Uncertainty Quantification was addressed by assessing the impact of varying amounts of historical patient data and different forecasting horizons on the models’ performance, measured by AUROC, AUPRC, sensitivity, and specificity. Furthermore, to assess our model’s reliability, we have incorporated several layers of uncertainty quantification:

1. Our model provides probabilistic estimations of the most likely future trajectories for disease parameters, conditioned on prior patient observations. This enables the quantification of confidence intervals for individual patients, offering a measure of uncertainty for possible future patient states.
2. As detailed in Methods section Annotation Model, our annotation model outputs a probability of disease progression at the individual patient level, which is used as a risk score. The distribution of this risk score among the patient cohort is illustrated in Fig. 3a and underpins the data presented in the rest of Fig. 3.
3. To further test the robustness of our model, we employed a 5-fold cross-validation approach. This method allowed us to quantify the variability of performance metrics across specific data partitions and evaluate the generalizability and robustness of our models. Additionally, the models from the five cross-validation folds of the CoMMpass dataset were externally validated on the GMMG-MM5 study dataset. This approach enables a comparison across models and provides a measure of how well the training data generalizes to the individual prediction for a given patient.

This comprehensive uncertainty quantification is designed to provide measures of confidence on distinct levels to ensure informed decision-making in a real-world setting.

### SHAP value computation

To approximate feature contributions for the outputs of our models, we calculated SHAP values using the DeepExplainer class^58,59^ of the SHAP python library^60^ (version 0.46.0). SHAP values are an estimation of classic Shapley values from game theory^61^. In a ML context they can be understood as a given feature’s contribution to the difference between the prediction by a model and a prior base value. According to best practices, the DeepExplainer was created using a randomly selected subset of 200 training data samples as the background dataset, while the individual SHAP values were calculated on basis of 60 randomly selected test data samples per cross-validation fold. In our analysis, we omitted the temporal dependency of the SHAP values, which naturally results from the structure of our data, by considering only the interaction between the last time step of the input sequence and the corresponding model output.

## Supplementary information

Supplementary Information

## Data Availability

The CoMMpass data is available upon registration in the MMRF Researcher Gateway at https://research.themmrf.org. The GMMG-MM5 study data is available upon reasonable request to hartmut.goldschmidt@med.uni-heidelberg.de.

## Code availability

We hosted a public GitHub repository at https://github.com/maximilianferle/predicting-progression-events-in-mm-from-routine-blood-work with all code used to produce the results of this study.
