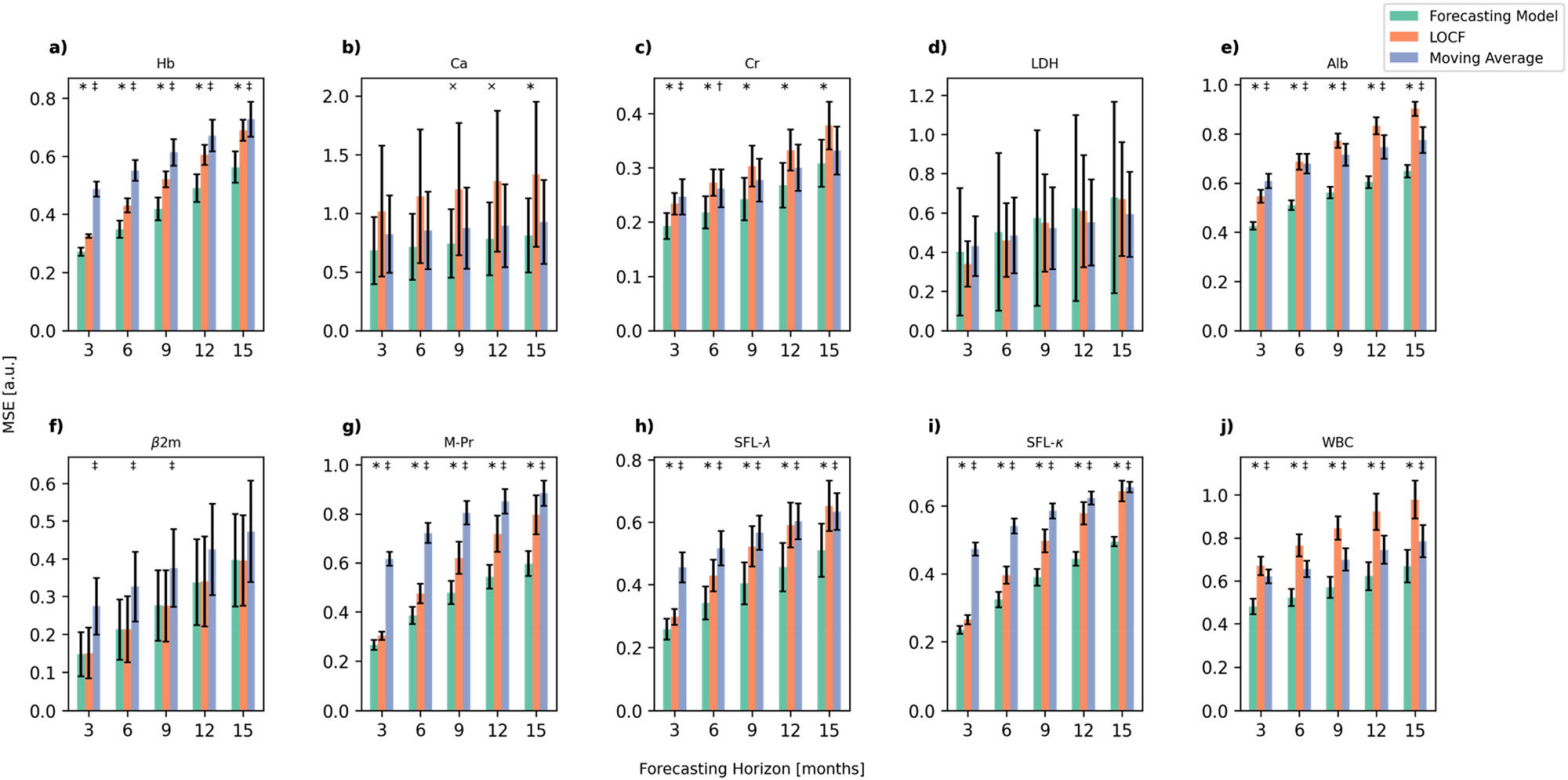# Publisher Correction: Predicting progression events in multiple myeloma from routine blood work

**DOI:** 10.1038/s41746-025-01719-7

**Published:** 2025-05-28

**Authors:** Maximilian Ferle, Nora Grieb, Markus Kreuz, Jonas Ader, Hartmut Goldschmidt, Elias K. Mai, Uta Bertsch, Uwe Platzbecker, Thomas Neumuth, Kristin Reiche, Alexander Oeser, Maximilian Merz

**Affiliations:** 1 Center for Scalable Data Analytics and Artificial Intelligence (ScaDS.AI) Dresden/Leipzig, Universität Leipzig, Leipzig, Germany; 2 Innovation Center Computer Assisted Surgery (ICCAS), University of Leipzig, Leipzig, Germany; 3 Department of Medical Bioinformatics, Fraunhofer Institute for Cell Therapy and Immunology, Leipzig, Germany; 4 Department of Hematology, Hemostaseology, Cellular Therapy and Infectiology, University Hospital of Leipzig, Leipzig, Germany; 5 Department of Internal Medicine V, University Hospital Heidelberg, Heidelberg, Germany; 6 National Center for Tumor Diseases (NCT), Heidelberg, Germany; 7 Institute for Clinical Immunology, University Hospital of Leipzig, Leipzig, Germany; 8 Synagen GmbH, Dresden, Germany; 9 Multiple Myeloma and Transplant and Cellular Therapy Services, Department of Medicine, Memorial Sloan Kettering Cancer Center, New York, NY USA

**Keywords:** Myeloma, Experimental models of disease, Cancer models

Correction to: *npj Digital Medicine* 10.1038/s41746-025-01636-9, published online 30 April 2025

In this article the wrong figure appeared as Fig. 1; the figure should have appeared as shown below. The original article has been corrected.