# Supplementary material for: Predicting progression events in multiple myeloma from routine blood work
Source: NPJ Digit Med. 2025 Apr 30;8:231. doi: 10.1038/s41746-025-01636-9 (PMC12043975; doi:10.1038/s41746-025-01636-9)
Supplement: Supplementary file 1 — Supplementary Information [file 41746_2025_1636_MOESM1_ESM.pdf]

Supplementary Material

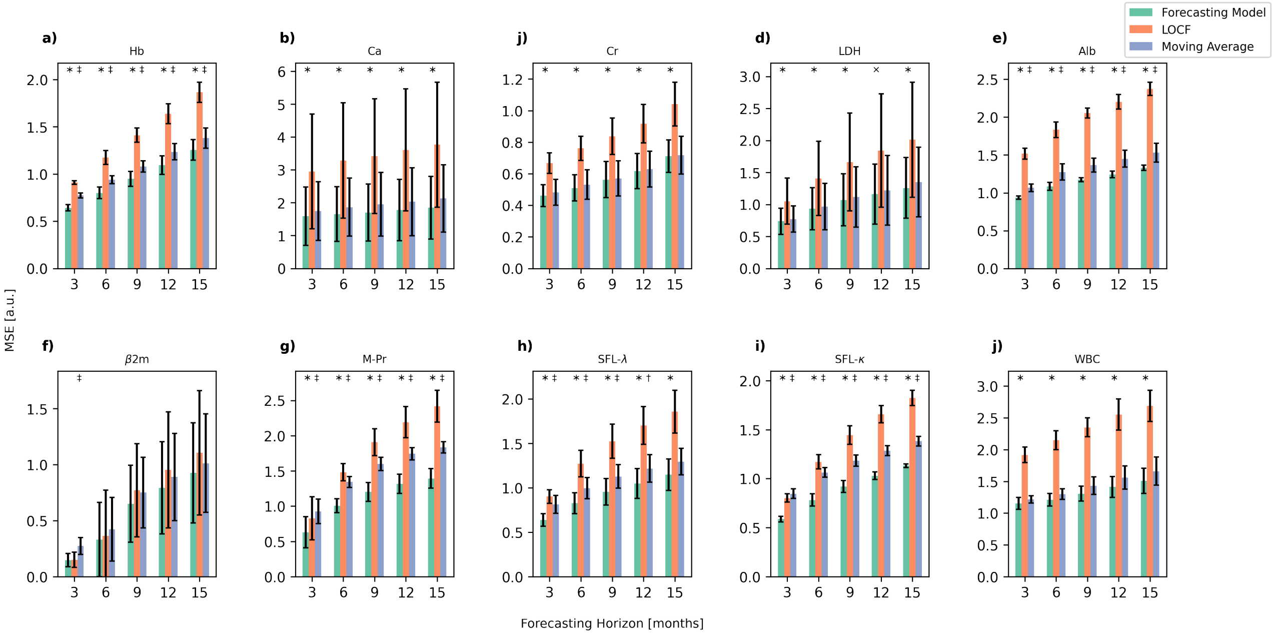

Supplementary Figure 1: **Focused Analysis on Extreme Changes in Forecasting Model Performance.** Analogous analysis to Figure 1, focusing on the most extreme 31.8% of changes. We filtered all instances where the changes of parameters fell within one standard deviation of all changes. Mean squared error (MSE) of three different forecasting approaches across various forecasting horizons. Error bars represent the standard deviation of the MSE obtained from cross-validation folds. The forecasting model (shown in green), last observation carried forward (LOCF, shown in orange), and moving average (MA, shown in blue) are compared. Each panel represents a different blood work parameter: (**a**) Hemoglobin (Hb), (**b**) Calcium (Ca), (**c**) Creatinine (Cr), (**d**) Lactate Dehydrogenase (LDH), (**e**) Albumin, (**f**) Beta-2-Microglobulin (β2m), (**g**) M-Protein (M-Pr), (**h**) Serum free light-chain lambda (SFL-λ), (**i**) Serum free light-chain kappa (SFL-κ), (**j**) White blood cells (WBC). Statistical significance is indicated by a cross and asterisk for comparisons between the forecasting model and LOCF, and a dagger and double dagger for comparisons between the forecasting model and MA, corresponding to p-values less than $p < 0.1$ and $p < 0.05$ respectively, based on a one-sided Mann-Whitney U test.

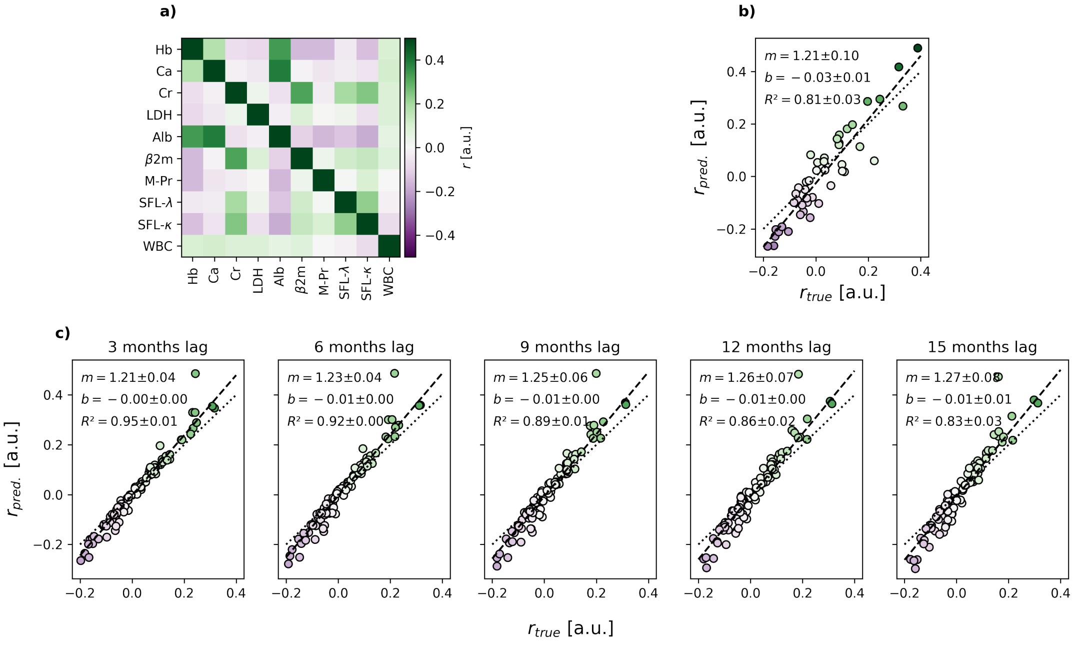

Supplementary Figure 2: **Statistical Moments of Blood Work Time Series Data Across Forecasting Horizons.** Statistical moments of the blood work time series data. The analysis includes (**a**) a correlation matrix of ten blood work parameters, calculated from ground truth patient data. Note that the colorbar is truncated to the range [-0.45, 0.45]. (**b**) Juxtaposition of the correlation coefficients from the actual data with those obtained from the forecasted data across varying forecasting horizons. Forecasting horizons ranged from 3 to 15 months in 3-month increments and were summarized to reflect the overall performance across all forecasting horizons. (**c**) Correlation coefficients for each pair of features across five distinct lag times: 3, 6, 9, 12, and 15 months. Observed data is juxtaposed against forecasted data. Dashed lines show the lines of best fit. Parameters are reported as mean ± standard deviation across cross-validation folds. Dotted lines show the expected lines of best fit for $m = 1$ and $b = 0$.

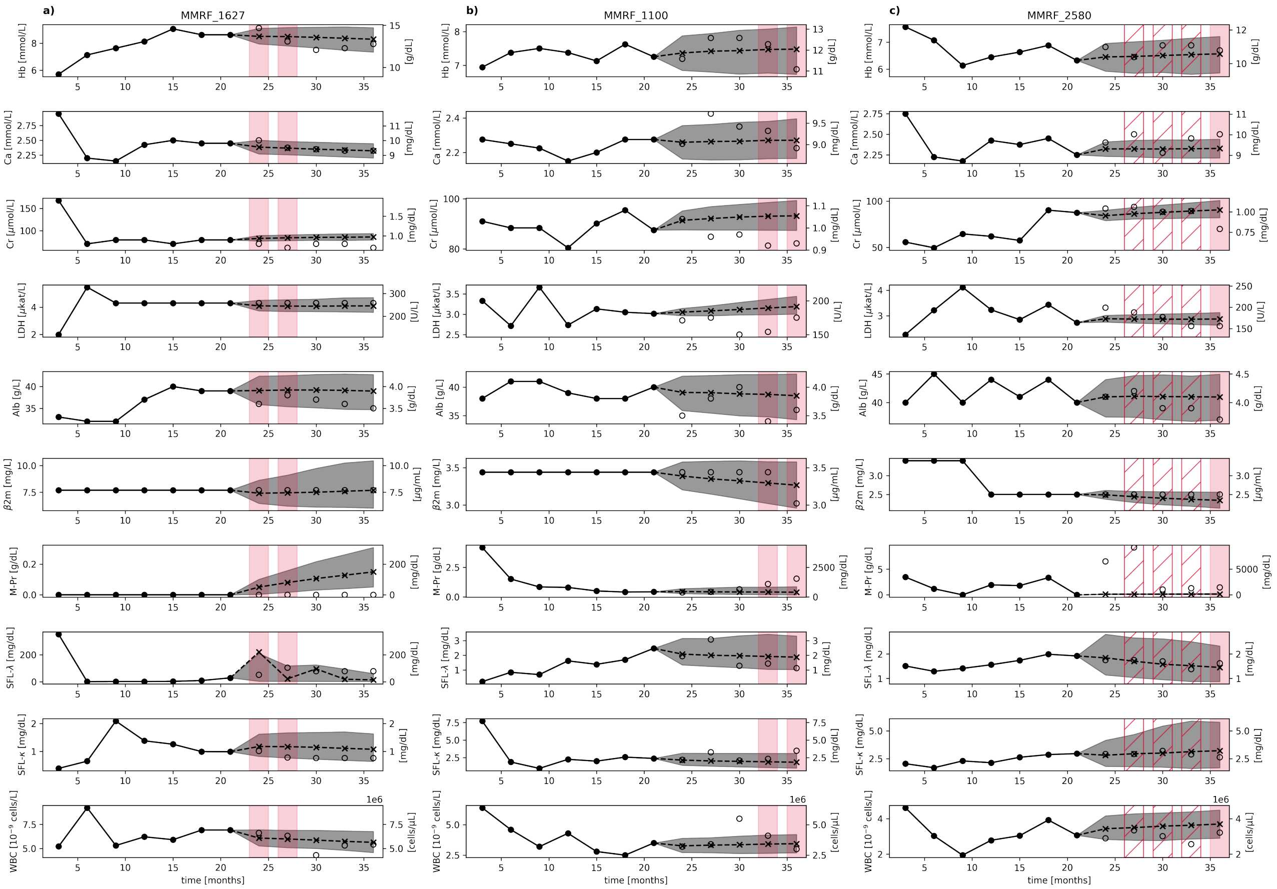

Supplementary Figure 3: **Extended Analysis of Forecasted and Annotated Progression Events.** Analogous analysis to that presented in Figure 2, focusing on the metamodel's performance. The metamodel was provided with the initial seven follow-ups, corresponding to 21 months of clinical data, to forecast the subsequent five follow-ups and annotate progression events, covering an additional 15 months. (**a**) Patient example where immediate progression events were correctly predicted. (**b**) Patient example where late progression events were correctly predicted. (**c**) Patient example where immediate progression events were missed but late progression events were correctly predicted. Dashed lines and crosses show forecasts, while circles show actual observations. Grey sleeves indicate the 95% confidence interval of the distribution of forecasted trajectories. Red shaded areas indicate instances where progression was correctly flagged, while hatched areas highlight where progression was missed.

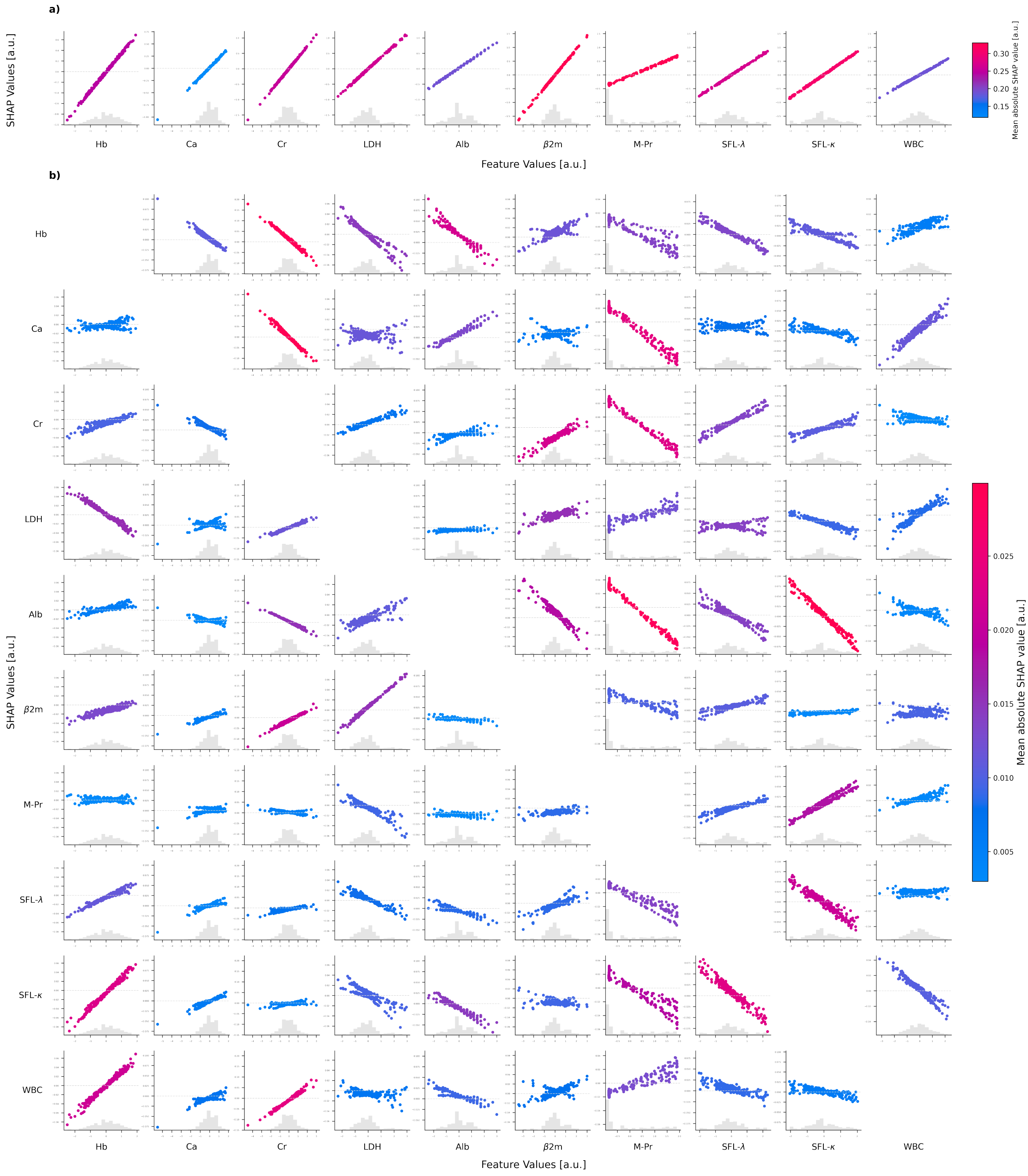

Supplementary Figure 4: **SHAP Value Analysis of Pairwise Effects Between Input and Output Features in the Forecasting Model.** SHAP value analysis of the pairwise effect of input features on output features of the forecasting model. Input feature values are shown on the x-axis with corresponding SHAP values (input feature's impact on the output feature) on the y-axis. The analysis includes (**a**) the impact of input features on themselves at the next timestep. (**b**) the impact of input features on other output features. Each row is labeled with the analyzed output feature and shows scatters of the corresponding SHAP values of the respective input feature (labeled per column).

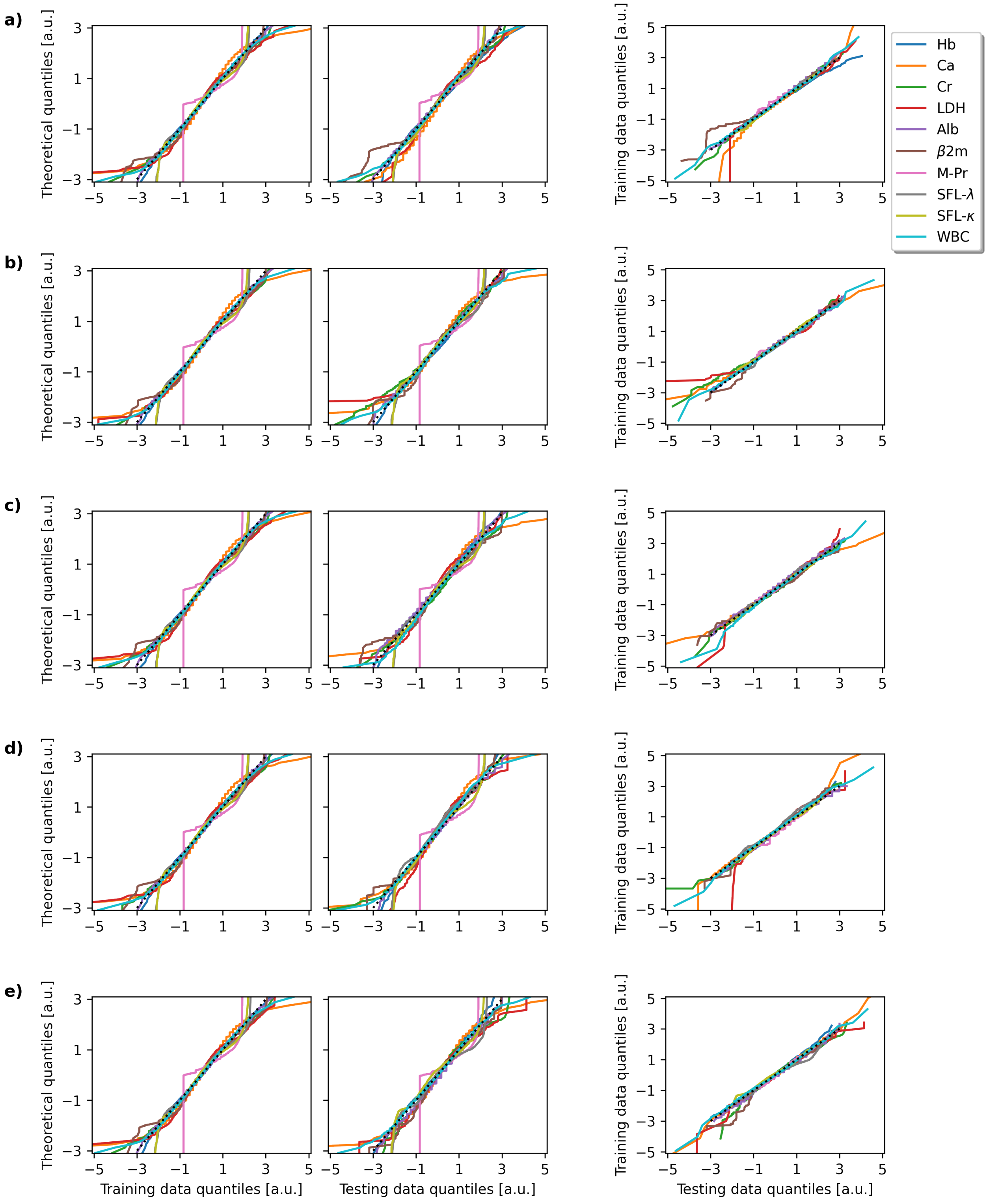

Supplementary Figure 5: **Quantile-Quantile Plots of Training and Testing Data After Power Transformation.** QQ-plots of the training and testing data after power transformation for various values of k (Cross-Validation Fold). The analysis includes $k = 1$ (**a**), $k = 2$ (**b**), $k = 3$ (**c**), $k = 4$ (**d**), $k = 5$ (**e**). Theoretical quantiles indicate quantiles of a normal distribution. Dotted lines indicate the range of the central 99.9% of the data.

Supplementary Table 1: Comparison of Pearson's r computed between actual blood work values and forecasts using our model, LOCF and moving average.

		**3 months**	**6 months**	**9 months**	**12 months**	**15 months**
**Hb**	Model	**0.82 ± 0.02**	**0.77 ± 0.02**	**0.72 ± 0.02**	**0.67 ± 0.03**	**0.63 ± 0.03**
	LOCF	0.81 ± 0.02	0.75 ± 0.03	0.70 ± 0.03	0.65 ± 0.04	0.61 ± 0.04
	Moving Average	0.72 ± 0.02	0.69 ± 0.03	0.65 ± 0.03	0.62 ± 0.03	0.59 ± 0.04
**Ca**	Model	**0.51 ± 0.08**	**0.44 ± 0.09**	**0.40 ± 0.08**	**0.36 ± 0.08**	**0.34 ± 0.08**
	LOCF	0.47 ± 0.12	0.39 ± 0.12	0.35 ± 0.1	0.33 ± 0.11	0.30 ± 0.1
	Moving Average	0.43 ± 0.09	0.38 ± 0.1	0.36 ± 0.12	0.36 ± 0.11	0.34 ± 0.1
**Cr**	Model	**0.89 ± 0.01**	**0.87 ± 0.01**	**0.85 ± 0.01**	**0.83 ± 0.01**	0.81 ± 0.01
	LOCF	0.87 ± 0.01	0.85 ± 0.01	0.83 ± 0.01	0.82 ± 0.01	0.79 ± 0.01
	Moving Average	0.86 ± 0.01	0.85 ± 0.01	0.84 ± 0.01	**0.83 ± 0.01**	**0.82 ± 0.02**
**LDH**	Model	0.82 ± 0.02	0.76 ± 0.03	0.71 ± 0.03	0.68 ± 0.03	0.64 ± 0.03
	LOCF	**0.83 ± 0.03**	**0.78 ± 0.04**	**0.74 ± 0.03**	**0.71 ± 0.04**	**0.68 ± 0.04**
	Moving Average	0.76 ± 0.06	0.73 ± 0.07	0.71 ± 0.07	0.70 ± 0.08	0.67 ± 0.08
**Alb**	Model	**0.70 ± 0.02**	**0.63 ± 0.03**	**0.58 ± 0.03**	**0.55 ± 0.03**	**0.52 ± 0.03**
	LOCF	0.68 ± 0.03	0.59 ± 0.03	0.54 ± 0.03	0.51 ± 0.03	0.48 ± 0.03
	Moving Average	0.58 ± 0.02	0.53 ± 0.03	0.51 ± 0.04	0.49 ± 0.04	0.47 ± 0.04
**β-2-Microglobulin**	Model	**0.92 ± 0.02**	0.88 ± 0.03	0.84 ± 0.03	0.81 ± 0.03	0.77 ± 0.03
	LOCF	**0.92 ± 0.02**	**0.89 ± 0.03**	**0.85 ± 0.03**	**0.82 ± 0.03**	**0.79 ± 0.03**
	Moving Average	0.86 ± 0.03	0.83 ± 0.03	0.80 ± 0.04	0.78 ± 0.04	0.75 ± 0.04
**M-Pr**	Model	**0.82 ± 0.01**	**0.72 ± 0.02**	**0.64 ± 0.03**	**0.58 ± 0.03**	**0.53 ± 0.03**
	LOCF	**0.82 ± 0.01**	**0.72 ± 0.02**	0.63 ± 0.03	0.57 ± 0.03	**0.53 ± 0.03**
	Moving Average	0.66 ± 0.01	0.59 ± 0.01	0.53 ± 0.02	0.50 ± 0.02	0.48 ± 0.02
**SFL-λ**	Model	**0.82 ± 0.01**	**0.75 ± 0.02**	**0.70 ± 0.02**	**0.66 ± 0.03**	**0.63 ± 0.03**
	LOCF	0.81 ± 0.01	0.73 ± 0.01	0.67 ± 0.02	0.64 ± 0.03	0.61 ± 0.04
	Moving Average	0.70 ± 0.01	0.66 ± 0.01	0.63 ± 0.01	0.61 ± 0.02	0.60 ± 0.03
**SFL-κ**	Model	**0.84 ± 0.01**	**0.76 ± 0.01**	**0.70 ± 0.02**	**0.66 ± 0.03**	**0.62 ± 0.04**
	LOCF	**0.84 ± 0.01**	0.75 ± 0.01	0.69 ± 0.02	0.64 ± 0.02	0.61 ± 0.03
	Moving Average	0.69 ± 0.04	0.64 ± 0.05	0.61 ± 0.05	0.59 ± 0.05	0.57 ± 0.05
**WBC**	Model	**0.68 ± 0.02**	**0.63 ± 0.02**	**0.59 ± 0.02**	**0.55 ± 0.02**	**0.52 ± 0.02**
	LOCF	0.63 ± 0.02	0.57 ± 0.02	0.53 ± 0.02	0.49 ± 0.02	0.47 ± 0.02
	Moving Average	0.60 ± 0.02	0.56 ± 0.03	0.53 ± 0.03	0.51 ± 0.03	0.49 ± 0.04

Supplementary Table 2: Pearson's r comparing the difference between the last known observation and the actual blood work values (Δ_{true}) to the difference between the last known observation and the forecast of our model (Δ_{model}).

	**3 months**	**6 months**	**9 months**	**12 months**	**15 months**
**Hb**	0.42 ± 0.03	0.44 ± 0.03	0.45 ± 0.03	0.44 ± 0.03	0.44 ± 0.03
**Ca**	0.57 ± 0.05	0.60 ± 0.05	0.61 ± 0.05	0.62 ± 0.05	0.62 ± 0.04
**Cr**	0.45 ± 0.04	0.47 ± 0.04	0.46 ± 0.04	0.46 ± 0.04	0.45 ± 0.04
**LDH**	0.35 ± 0.07	0.39 ± 0.07	0.40 ± 0.06	0.40 ± 0.04	0.41 ± 0.05
**Alb**	0.48 ± 0.02	0.52 ± 0.02	0.53 ± 0.02	0.53 ± 0.02	0.54 ± 0.02
β**-2-Microglobulin**	0.26 ± 0.05	0.25 ± 0.03	0.25 ± 0.03	0.26 ± 0.02	0.26 ± 0.03
**M-Pr**	0.38 ± 0.02	0.44 ± 0.01	0.48 ± 0.02	0.50 ± 0.01	0.51 ± 0.01
**SFL-**λ	0.40 ± 0.02	0.47 ± 0.02	0.49 ± 0.02	0.49 ± 0.02	0.48 ± 0.03
**SFL-**κ	0.37 ± 0.03	0.44 ± 0.01	0.47 ± 0.01	0.49 ± 0.01	0.49 ± 0.02
**WBC**	0.53 ± 0.02	0.56 ± 0.02	0.57 ± 0.02	0.57 ± 0.02	0.56 ± 0.02
